# Miro1 Is a Calcium Sensor for Glutamate Receptor-Dependent Localization of Mitochondria at Synapses

**DOI:** 10.1016/j.neuron.2009.01.030

**Published:** 2009-02-26

**Authors:** Andrew F. MacAskill, Johanne E. Rinholm, Alison E. Twelvetrees, I. Lorena Arancibia-Carcamo, James Muir, Asa Fransson, Pontus Aspenstrom, David Attwell, Josef T. Kittler

**Affiliations:** 1 Department of Neuroscience, Physiology, and Pharmacology, University College London, Gower Street, London WC1E 6BT, UK; 2 Ludwig Institute for Cancer Research, Biomedical Center, Uppsala University, SE-751 24, Sweden

**Keywords:** MOLNEURO, CELLBIO, SIGNALING

## Abstract

Energy use, mainly to reverse ion movements in neurons, is a fundamental constraint on brain information processing. Trafficking of mitochondria to locations in neurons where there are large ion fluxes is essential for powering neural function. Mitochondrial trafficking is regulated by Ca^2+^ entry through ionotropic glutamate receptors, but the underlying mechanism is unknown. We show that the protein Miro1 links mitochondria to KIF5 motor proteins, allowing mitochondria to move along microtubules. This linkage is inhibited by micromolar levels of Ca^2+^ binding to Miro1. With the EF hand domains of Miro1 mutated to prevent Ca^2+^ binding, Miro1 could still facilitate mitochondrial motility, but mitochondrial stopping induced by glutamate or neuronal activity was blocked. Activating neuronal NMDA receptors with exogenous or synaptically released glutamate led to Miro1 positioning mitochondria at the postsynaptic side of synapses. Thus, Miro1 is a key determinant of how energy supply is matched to energy usage in neurons.

## Introduction

The brain is 2% of the body's weight, but consumes 20% of the body's resting energy production. Energy is used mainly on reversing the ion influxes underlying synaptic and action potential signaling in neurons (Attwell and Laughlin, 2001). This high energy consumption is a constraint on the information processing carried out by the brain and requires that at the circuit level the neural wiring and information codes employed by neurons be highly energy efficient (Laughlin and Sejnowski, 2003). The limitations imposed by energy use on brain function, and the large size of many neurons (which precludes rapid diffusion of ATP from one end of the cell to the other), also imply that at the single-cell level energy production must be spatially matched to local energy usage. Since most brain ATP is generated by mitochondria, this implies that mitochondria must be located spatially close to the sites of the ion influxes that generate synaptic and action potentials.

In neurons, mitochondria are highly dynamic, exhibiting activity-induced alterations in transport and distribution (Chen and Chan, 2006; Hollenbeck and Saxton, 2005). The active concentration of mitochondria in specific neuronal regions such as growth cones and synapses (Chang et al., 2006; Kang et al., 2008; Li et al., 2004) is important for correct neuronal function and development (Guo et al., 2005; Kang et al., 2008; Tang and Zucker, 1997). Mutations in proteins regulating mitochondrial dynamics compromise synaptic function and plasticity (Li et al., 2004; Verstreken et al., 2005), and defective mitochondrial trafficking is implicated in neurological and neurodegenerative disease (Alexander et al., 2000; Baloh et al., 2007; Chan, 2006; Chen and Chan, 2006).

Mitochondrial motility is inhibited by raised levels of Ca^2+^ (Yi et al., 2004). In neurons, calcium entry through ionotropic glutamate receptors reduces mitochondrial motility (Chang et al., 2006; Rintoul et al., 2003). Ca^2+^-dependent control of mitochondrial motility is therefore likely to be important for determining the spatial localization of mitochondria under normal conditions and may also cause pathological alterations in mitochondrial function during glutamate-evoked excitotoxic neuronal injury. However, the identity of the Ca^2+^ sensor and the mechanisms that mediate Ca^2+^-dependent regulation of mitochondrial trafficking are unknown.

**Figure 1:**
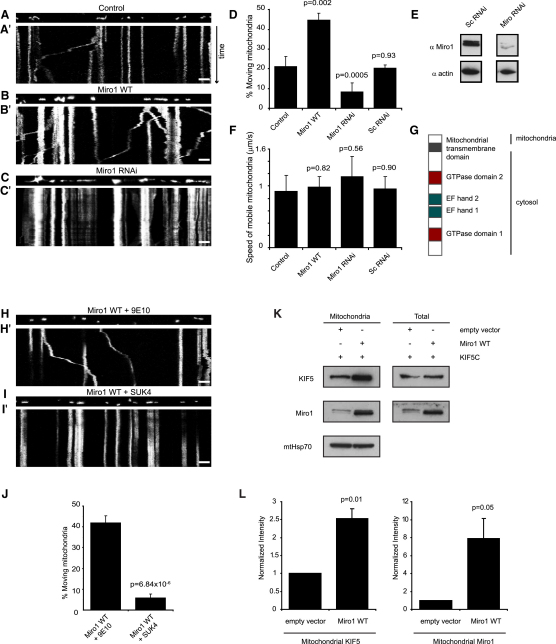
Altering Miro1 Expression Affects Mitochondrial Mobility via an Interaction with KIF5 Neurons were transfected with mtdsred2 as well as Miro1 GFP or shRNAi to Miro1, or mtdsred2 alone, 2–3 days before being imaged at DIV 12–14. (A–C) Static image of a dendrite at time = 0 in mtdsred2-transfected cell (A), mtdsred2- and Miro1-transfected cell (B), and mtdsred2- and Miro1 RNAi-transfected cell (C). (A′–C′) Kymographs showing increased mitochondrial movement in a neuronal dendrite upon Miro1 expression (B′) and decreased mitochondrial movement upon RNAi expression (C′) compared to controls (A′). Height, 2 min (time increases down the page); scale bar, 10 μm. (D) Percentage of mitochondria moving in dendrites of control cells (n = 8 dendrites, 295 mitochondria), cells expressing Miro1 (n = 8 dendrites, 144 mitochondria), cells expressing Miro1 RNAi (n = 9 dendrites, 569 mitochondria), and cells expressing a scrambled control RNAi (n = 7 dendrites, 412 mitochondria). Error bars here and throughout represent the standard error of the mean. p values compare with the control bar. (E) Western blot showing specific knockdown of Miro1 in cultured cortical neurons using Miro1 RNAi. Actin used as a control for loading shows no change. (F) Average velocity of moving mitochondria in dendrites of control cells, cells expressing Miro1, and cells expressing Miro1 RNAi. (G) Schematic of the primary structure of Miro1. (H–I′) Static images and kymographs showing mitochondrial movement through dendrites transfected with Miro1 GFP and transduced with either control (9E10) antibody (H) or a function-blocking KIF5 motor (SUK4) antibody (I). Height, 2 min; scale bar, 10 μm. (J) Percentage of mitochondria moving in dendrites of control 9E10-transduced cells (n = 8 dendrites, 386 mitochondria) and SUK4-transduced cells (n = 11 dendrites, 211 mitochondria). (K) COS7 cells were transfected with KIF5C and either an empty vector or Miro1. Mitochondria were isolated, and blots of the mitochondrial fraction of the cells, and of the total cell lysate, were probed for KIF5C, Miro1, and (as a reference mitochondrial protein) mtHsp70. In the mitochondrial fraction the amount of KIF5C (and of Miro1) is increased by Miro1 expression (compared to empty vector), but the total level of KIF5C is unaltered. (L) Quantification of four replicates of the data in (K), with the levels of KIF5C and Miro1 normalized to the level of the reference protein mtHsp70 in the mitochondrial fraction.

Mitochondrial trafficking in neurons is mediated by kinesin motors (Hirokawa and Takemura, 2005; Hollenbeck, 1996; Pilling et al., 2006). Various candidates for linking mitochondria to trafficking machinery have been proposed, including syntabulin (Cai et al., 2005) and syntaphilin (Kang et al., 2008), but these lack obvious coupling to signaling cascades that could regulate the trafficking. Several GTPases regulate the distribution of neuronal mitochondria, including Drp1, OPA1, Mitofusins (Alexander et al., 2000; Choi et al., 2006; Frank et al., 2001; Li et al., 2004) and the Mitochondrial Rho GTPase (Miro) (Guo et al., 2005). In *Drosophila*, dMiro forms a transport complex with the kinesin adaptor Milton (Glater et al., 2006; Stowers et al., 2002), and in *dMiro* mutants, mitochondria are not transported into neuronal processes but remain clustered in the neuronal somata (Guo et al., 2005). Miro proteins contain a transmembrane domain locating them to the outer mitochondrial membrane, with two GTPase domains and two Ca^2+^-sensing EF hand domains protruding into the cytoplasm (Figure 1G; Fransson et al., 2003). Miro therefore has properties suitable for coupling cytoplasmic Ca^2+^ sensing to mitochondrial trafficking (Rice and Gelfand, 2006).

Here we investigate the role of Miro1 in regulating mitochondrial mobility in mammalian neuronal dendrites. We show that Miro1 mediates trafficking of mitochondria with KIF5 motor proteins, linkage of mitochondria to KIF5 motors by Miro1 is inhibited by Ca^2+^ binding to Miro1's EF hands, and calcium influx evoked by glutamate receptor activation causes mitochondria to accumulate at synapses in a Miro1-dependent manner. Miro1 can therefore regulate mitochondrial trafficking in response to changes of Ca^2+^ concentration within dendrites and provides a mechanism that matches energy supply to energy use at different subcellular locations of neurons.

## Results

### Miro1 Regulates Mitochondrial Motility

Miro1 is localized to mitochondria in mammalian cell lines (Fransson et al., 2003, 2006) and *Drosophila* neurons (Guo et al., 2005), but its location in mammalian neurons is unknown. We carried out live-cell confocal microscopy of rat hippocampal neurons expressing Miro1-GFP and mitochondrially targeted red fluorescent protein (mtdsred2; labeling of mitochondria with mtdsred2 did not affect the percentage of mitochondria that were moving: Figures S1A and S1B available online). Miro1-GFP in neuronal processes showed essentially complete overlap with mtdsred2, as would be predicted for a mitochondrially localized protein (Figures S1C and S1D). Dendritic processes were then selected by morphological analysis and/or MAP2 staining (Figure S2). By imaging over time, motile and stationary mitochondria in dendrites were visualized using kymographs (Figures S1A, S1E, and S1F) that were created by projecting sequential line scans through a process of interest onto the y axis (Miller and Sheetz, 2004). Stationary mitochondria are seen as straight lines and moving mitochondria as diagonal lines. Using this technique, Miro1 GFP could be seen to be present in both moving and stationary mitochondria in dendrites, in contrast to other mitochondrial adaptors such as syntaphilin, which is specifically localized to a stationary pool of mitochondria (Kang et al., 2008).

We investigated the effect of altering Miro1 expression level on the dynamics of mitochondrial movement (Figure 1) in hippocampal neurons cotransfected with mtdsred2 to visualize mitochondria and either wild-type (WT) Miro1 to increase Miro1 expression levels or shRNAi to reduce Miro1 expression levels. In control neurons, the percentage of mitochondria that were moving in dendrites, assessed over a 2 min period, was around 20%, similar to previous observations (Overly et al., 1996). Expression of Miro1 caused an increase in the percentage of mitochondria that were moving in dendrites (41.8% ± 2.5%, p = 0.002 compared with control; Figures 1A, 1B, and 1D and Movie S1). In contrast, using shRNAi to specifically knock down Miro1 (Figures 1C–1E) resulted in a much smaller fraction of mitochondria being mobile in dendrites compared to scrambled control transfected cells (8.9% ± 0.6%, p = 0.0005; Figures 1C and 1D). This effect of Miro1 knockdown was replicated using a shRNAi targeting a different part of the Miro1 sequence and was rescued by expression of human Miro1 (which is resistant to knockdown by an RNAi sequence specific to rat: Figure S3).

The density of mitochondria along dendrites was not altered by these manipulations (the number of mitochondria per micron was 0.14 ± 0.01 (in eight dendrites) in control conditions, 0.15 ± 0.02 (n = 9) with Miro1 transfected (p = 0.31 compared with control), 0.16 ± 0.01 (n = 10) with shRNAi to Miro1 (p = 0.33 compared with control), and 0.16 ± 0.01 (n = 6) with scrambled shRNAi (p = 0.21 compared with control). Furthermore, if we analyzed only moving mitochondria, their average velocity did not change upon Miro1 expression. In control neurons it was 0.91 ± 0.26 μm.s^−1^, in Miro1-expressing neurons it was 0.98 ± 0.17 μm.s^−1^ (p = 0.82), and in Miro1 RNAi-expressing neurons it was 1.15 ± 0.33 μm.s^−1^ (p = 0.56 compared with control; Figure 1F). These values fall into the range previously reported for mitochondrial motility in neurons (Schuman and Chan, 2004). Thus, increasing Miro1 expression only increases the fraction of mitochondria engaged in the mobile phase rather than providing alternative pathways for more rapid movement. This effect of increasing Miro1 occurred equally for mitochondria moving along dendrites toward the soma and for mitochondria moving away from the soma (Figure S4).

### Altered Transport by Miro1 Is via an Interaction with KIF5 Motor Proteins

Mitochondrial transport has been shown to be dependent on kinesin family 1 (KIF5) motor proteins (Tanaka et al., 1998; Hirokawa and Takemura, 2005). To determine whether the increase in mitochondrial motility that we observed upon Miro1 expression was dependent on enhanced coupling to KIF5, we introduced into neurons transfected with Miro1 a KIF5 function-blocking antibody (SUK4; Ingold et al., 1988; Jaulin et al., 2007) that does not inhibit myosin- or dynein-based motility (Bi et al., 1997; Lane and Allan, 1999), or a control antibody (9E10). This was done by complexing the antibody with a membrane-permeant carrier peptide (Figure S5; Kittler et al., 2006; Morris et al., 2001). The KIF5 function-blocking antibody strikingly lowered the fraction of moving mitochondria in neurons transfected with Miro1 (Figures 1H–1J: 5.9% ± 1.8% of mitochondria were moving in Miro1-transfected neurons treated with SUK4 antibody, compared to 42.0% ± 5.2% in Miro1-transfected neurons treated with control 9E10 antibody, p = 6.8 × 10^−6^), confirming that Miro1-dependent facilitation of mitochondrial transport is due to Miro1 increasing the fraction of mitochondria that are coupled to KIF5 motor proteins. Consistent with this, in COS7 cells we found that overexpressing Miro1 led to an increased association of KIF5 with mitochondria (Figures 1K and 1L: a demonstration of a Miro1-mediated alteration in mitochondrial trafficking being associated with an alteration in the amount of KIF5 bound to mitochondria in neurons will be presented below).

### Miro1 Binds Directly to KIF5 Motors

**Figure 2:**
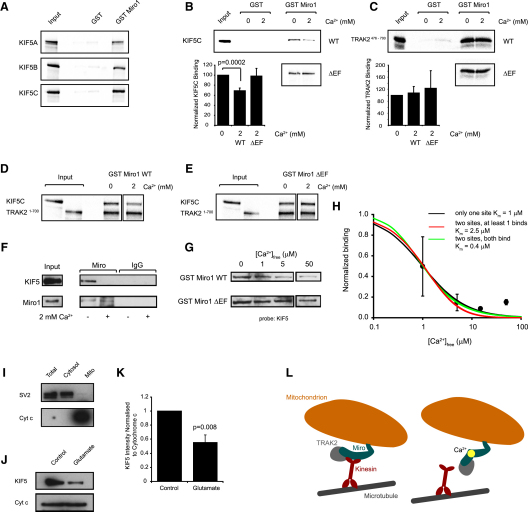
Ca^2+^-Dependent Binding of Miro1 to KIF5 Motors In Vitro and In Situ (A–E) The kinesin subunits KIF5A, KIF5B, or KIF5C, and TRAK2 fragments (476–700 or 1–700) were translated in vitro and labeled with ^35^S-methionine. The resulting protein was subjected to pull-down assay with GST-Miro1 or GST-Miro1-ΔEF or with GST alone as control, separated by SDS-PAGE, and radioactivity was detected using a phosphor storage screen. Input represents 10% of input used in each experiment. (A) Pull-down assay demonstrating that Miro1 binds directly to ^35^S-labeled KIF5A, KIF5B, and KIF5C (with no other proteins in the system). No added Ca^2+^ present. GST alone did not bind KIF motors. (B) Repeat of (A) in different [Ca^2+^]. High [Ca^2+^] inhibits Miro1 binding to KIF5C, and this inhibition is absent when Miro1's EF hands are mutated. (C) Pull-down assay demonstrating that Miro1 binds directly to TRAK2 (amino acids 476–700), with no other proteins in the system, in a Ca^2+^-independent manner. (D) Pull-down assay with only KIF5C, Miro1, and TRAK2 (amino acids 1–700) in the system. As in (B) and (C), Miro1 binds to KIF5C in a Ca^2+^-dependent manner, but binds to TRAK2 in a Ca^2+^-independent manner. (E) As in (D), but using the EF hand mutant of Miro1: binding of Miro1-ΔEF to KIF5C is Ca^2+^ independent. (F) Coimmunoprecipitation showing that binding of endogenous Miro1 and KIF5 motors is Ca^2+^ sensitive. (G and H) Binding of Miro1 to KIF5 is sensitive to calcium in the physiological range, and this sensitivity is occluded by a mutation in the EF hand domains of Miro1 (Miro1 ΔEF). Solubilized brain homogenates were subjected to a GST pull-down assay with either GST-Miro1 WT or GST-Miro1-ΔEF in the presence of varying concentrations of [Ca^2+^]_free_ as shown (G). Equal protein loading was visualized using Ponceau stain. The GST-Miro1 WT-KIF5 interaction was blocked by calcium, while the GST-Miro1 ΔEF interaction showed little calcium sensitivity. (H) shows quantification of data as in (G), as a function of [Ca^2+^]_free_ (normalized to data in zero Ca^2+^; n = 3 for 1 μM, 2 for 5, 50 μM, 1 for 15 μM). Curves have the forms (with c = [Ca^2+^], K = K_m_ of site): K/(c + K) if there is only one EF hand binding Ca^2+^ to inhibit the interaction (black line); {K/(c + K)}^2^ if two EF hands of identical K_m_ can bind Ca^2+^ and the interaction with KIF5 is inhibited if one or both of them bind Ca^2+^ (red); and 1 − {c/(c + K)}^2^ if both EF hands have to bind Ca^2+^ to block the interaction (green). Best-fit K_m_ values are shown in the inset. (I–K) Glutamate evokes dissociation of KIF5 from mitochondria in brain slices. (I) After fractionation of brain slice lysate, essentially all cytochrome c is in the mitochondrial fraction, and none in the vesicle fraction (probed with antibody to the vesicle protein SV2). (J) Glutamate treatment of brain slices (100 μM for 10 min, with 1 μM glycine) decreases the amount of KIF5 in the mitochondrial fraction. (K) Quantification of data as in (J) on eight slices from six rats. (L) Schematic mechanism to explain the data. Miro1 links mitochondria to KIF5 motors, and a glutamate-evoked rise of [Ca^2+^] dissociates Miro1 from KIF5. TRAK2 remains bound to Miro1 even when [Ca^2+^] is high.

Miro1 could facilitate mitochondrial trafficking by acting as a direct linker between mitochondria and KIF5 motors, or there may be an extra coupling protein involved: a role suggested for the protein Milton in *Drosophila* (Glater et al., 2006). There is no direct homolog of Milton in mammals, but the GRIF-1/TRAK2 protein (Brickley et al., 2005) has 22% identity and 35% similarity. To test whether Miro1 can bind directly to KIF5 motor proteins, we constructed a GST fusion protein of Miro1 and used it to pull down in vitro translated radiolabeled (with ^35^S-methionine) versions of each of KIF5A, KIF5B, and KIF5C. With no other proteins in the system (and with no added Ca^2+^), each of these motor proteins was found to bind directly to Miro1 (Figure 2A). Thus, Miro1 does not need TRAK2 present to bind to KIF5 motors in vitro.

### The Miro1-Kinesin Interaction Is Calcium Sensitive In Vitro

Because Miro1 contains Ca^2+^-sensing EF hand domains, and our results demonstrate that Miro1 facilitates mitochondrial motility in a KIF5-dependent manner, we assessed whether Miro1 could form a protein complex with KIF5 that was regulated by Ca^2+^. We tested this not only for Miro1, but also for a mutant version of Miro1 (Miro1 ΔEF) containing mutations (E208K and E328K) in the Miro1 EF hand Ca^2+^-binding motifs.

Repeating the pull-down assay in the presence of 2 mM Ca^2+^, we found that Ca^2+^ significantly inhibited the binding of Miro1 to KIF5 motors (p = 0.0002; Figure 2B) and that this inhibition was not present when the EF hand mutant of Miro1 was used (p = 0.92; Figure 2B). In contrast, binding of in vitro translated and radiolabeled TRAK2 to Miro1 (with no other proteins in the system) was independent of Ca^2+^ concentration (Figure 2C). In case the presence of TRAK2 and Miro1 together altered how Miro1 interacts with KIF motors, we then extended this in vitro GST fusion pull-down assay to test the Ca^2+^ dependence of Miro1 binding to KIF5C and to TRAK2 with all three proteins present together. As before, the binding of Miro1 to KIF5C was inhibited by Ca^2+^, and this inhibition was not seen for the EF hand mutant of Miro1, while the binding of Miro1 to TRAK2 was not affected by Ca^2+^ (Figures 2D and 2E).

Consistent with this, using a ^45^Ca^2+^ overlay assay (Maruyama and Nonomura, 1984), we found that GST-Miro1 WT bound ^45^Ca^2+^, confirming that Miro1 is a Ca^2+^-binding protein (as predicted from its containing two EF hand motifs), while Ca^2+^ binding was significantly reduced (p = 0.0017) in the GST-Miro1 ΔEF mutant (Figures S6F–S6H).

### The Miro1-Kinesin Interaction Is Calcium Sensitive In Situ

Having demonstrated that Miro1 and KIF5 motors can interact in a Ca^2+^-dependent manner in a reduced in vitro system containing only these proteins, we then used coimmunoprecipitation experiments to investigate the calcium dependence of the formation of Miro1-KIF5 motor protein complexes in situ in rat brain. KIF5 motors (detected using a pan-KIF5 antibody) specifically coprecipitated with Miro1 antibody and not with control IgG (Figure 2F). To determine whether the Miro1-KIF5 interaction was sensitive to Ca^2+^, we performed the immunoprecipitation in the presence of 2 mM Ca^2+^. While similar levels of Miro1 were precipitated under conditions of both high and low [Ca^2+^], high [Ca^2+^] completely abolished the coprecipitation of KIF5 motor proteins (Figure 2F), demonstrating that formation of the Miro1-KIF5 protein complex is inhibited by Ca^2+^.

Using GST pull-down interaction assays from rat brain, we then tested whether the effect of Ca^2+^ occurs in the physiological range of [Ca^2+^]_free_ and whether it is blocked in the Miro1 ΔEF mutant (Figures 2G and 2H). GST-Miro1 WT showed a strong calcium dependence to its interaction with KIF5 motors, with the interaction being halved at an IC_50_ of ∼1 μM [Ca^2+^] and decreased to 15% at 5 μM [Ca^2+^]_free_ compared to levels in 0 [Ca^2+^]. If we assume that only one of Miro's EF hands is involved in blocking the interaction with KIF motors, so that there is a first-order dependence of the inhibition on [Ca^2+^]_free_, fitting the data in Figure 2H gives a K_m_ for the block of 1 μM. If instead it is assumed that the two EF hands bind Ca^2+^ independently with the same affinity (i.e., ignoring possible cooperativity) and that mitochondrial transport is inhibited by binding of Ca^2+^ to either of them or that Ca^2+^ needs to bind to both of them to block transport, then the predicted K_m_ becomes 2.5 μM or 0.4 μM, respectively (the resulting fits are shown in Figure 2H; the scatter in the data precludes using these plots to decide on which binding model fits the data best, but it is clear that for all the models, as for the experimental data, binding is largely abolished by 5 μM Ca^2+^).

GST-Miro1 ΔEF showed little calcium sensitivity to its interaction with KIF5 (Figure 2G), with the binding in 5 μM [Ca^2+^] remaining at 87% of the level in 0 [Ca^2+^] (significantly different from the value for wild-type Miro1, p = 0.021). Fitting a curve assuming just one Ca^2+^-binding site as for Miro1 WT gave a K_m_ of 69 μM (data not shown), i.e., 70-fold higher than for WT Miro1, confirming that the calcium dependence of the Miro1-KIF5 interaction is mediated through the EF hand domains of Miro1.

The pull-down experiments in Figure 2G were carried out without Mg^2+^ present. We have not looked at the effect of Mg^2+^ on the Miro1-KIF5 interaction, but many EF hands bind some Mg^2+^ when [Ca^2+^] is low (Gifford et al., 2007), and changes in [Mg^2+^] could alter the apparent affinity of Miro1 for Ca^2+^. Nevertheless, the fact that, as described below, removing Ca^2+^ from the extracellular solution abolishes the Miro1-mediated stopping of mitochondrial movement that is produced by activation of glutamate receptors (Figures 5C and S6D) suggests that the physiological regulator of the Miro1-KIF5 interaction is a rise of [Ca^2+^]_i_.

### Miro1 EF Hands Are the Calcium Sensor for Glutamate Receptor-Dependent Mitochondrial Stopping

**Figure 3:**
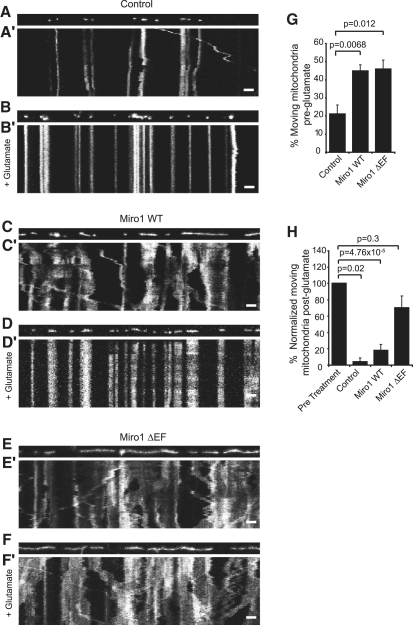
Miro1 Mediates Glutamate-Dependent Mitochondrial Stopping (A–F) Neurons transfected with Miro1 WT and mtdsred2, Miro1 ΔEF and mtdsred2, or mtdsred2 alone were treated with 30 μM glutamate and 1 μM glycine for 10 min. Dendrites were imaged at times −3 to −1 min and 10–12 min with respect to the start of glutamate application. Kymographs (A′–F′) show mitochondrial movement in control (A and B), Miro1 WT-transfected (C and D), and Miro1 ΔEF-transfected (E and F) dendrites before (A, C, and E) and after (B, D, and F) glutamate treatment. Height, 2 min (time increases down the page); scale bars, 10 μm. (G) Percentage of mitochondria moving before glutamate treatment in dendrites of control cells (n = 8 dendrites, 354 mitochondria), Miro1 WT-transfected cells (n = 8 dendrites, 257 mitochondria), and Miro1 ΔEF-transfected cells (n = 8 dendrites, 253 mitochondria). (H) Percentage of mitochondria moving after glutamate treatment, normalized to the percentage of mitochondria moving before treatment.

The above data suggest that Miro1 is a Ca^2+^ sensor that could transduce Ca^2+^ signals into an inhibition of mitochondrial coupling to KIF5-mediated transport. Transfection of Miro1 ΔEF into neurons produced an increase in the number of moving mitochondria similar to that observed for transfection of Miro1 WT (46.0% ± 4.7% of mitochondria were moving in Miro1 ΔEF-transfected neurons compared to 44.8% ± 3.4% in neurons transfected with Miro1 WT and 21.1% ± 5.1% for control neurons; Figure 3A, 3C, 3E, and 3G). Thus, mutation of the Ca^2+^-binding domains of Miro1 has no effect on its ability to recruit mitochondria to the KIF5 transport pathway under basal conditions. This lack of effect suggests that the resting calcium concentration does not affect Miro1-dependent trafficking, consistent with the IC_50_ for the interaction (∼1 μM, see above) being 20-fold higher than the resting [Ca^2+^]_i_ (∼50 nM: Maravall et al., 2000).

To investigate the role of Miro1 in Ca^2+^-dependent control of mitochondrial movement, we first studied mitochondrial stopping observed in neurons upon activation of ionotropic glutamate receptors (Chang et al., 2006; Rintoul et al., 2003). Hippocampal neurons were imaged using live-cell time-lapse confocal microscopy under steady-state conditions and after activation of ionotropic glutamate receptors. In control cells expressing only mtdsred2, perfusion of 30 μM glutamate (with 1 μM glycine to allow activation of NMDA receptors) for 10 min resulted in an almost complete cessation of mitochondrial movement in dendrites (the number of moving mitochondria was reduced by 95.7% ± 4.3% compared to the number moving prior to glutamate application, p = 0.02, Figures 3B′ and 3H; see Movie S2). This effect was dependent on extracellular calcium, as stopping of mitochondria was not observed upon glutamate receptor activation in 0 [Ca^2+^]_e_ (there was a 28% ± 9% reduction in movement in 0 [Ca^2+^]_e_ on addition of glutamate [not significant, p = 0.67], which was significantly different from the large reduction seen with extracellular Ca^2+^ present [p = 0.001]; Figure S6A–S6D). In contrast, the movement of GFP-synaptophysin-labeled vesicles was not affected by glutamate treatment (glutamate treatment reduced moving vesicles by only 11% ± 7%, p = 0.23; Figure S7 and Movie S3), showing that glutamate-dependent mitochondrial stopping is not the result of a global inhibition of the trafficking of all types of organelle.

In neurons expressing Miro1 WT, glutamate treatment reduced the number of moving mitochondria by 81.9% ± 7.3% (p = 5 × 10^−5^; Figures 3C, 3D, 3G, and 3H and Movie S4) just as for control neurons expressing mtdsred2 alone.

In marked contrast to control neurons or neurons transfected with Miro1 WT, neurons expressing Miro 1 ΔEF showed essentially no glutamate-dependent modulation of mitochondrial trafficking. The fraction of mitochondria moving was not significantly reduced by glutamate (reduced by 30.0% ± 14.5%, p = 0.3, with Miro1 ΔEF compared to 95.7% ± 4.3% in control neurons). Thus, Miro1 ΔEF significantly blocked the glutamate-mediated reduction in movement (p = 0.005; Figures 3E–3H and Movie S5).

As large increases in glutamate level can be excitotoxic, we tested whether similar rises in [Ca^2+^] without glutamate could also cause the same effect. The L-type calcium channel agonist FPL-64176, when coupled with 50 mM KCl depolarization, has been shown to raise calcium to levels similar to those produced by 30 μM glutamate, while not causing excitotoxic cell death (Li et al., 2001). Treatment of neurons with FPL-64176 and 50 mM KCl for 10 min caused the same effect on mitochondrial movement as glutamate treatment (Figure S6E), with transport in control and Miro1 WT cells being greatly reduced (by 64.1% ± 8.2%, p = 0.0004, and 80.0% ± 5.9%, p = 2.4 × 10^−6^, respectively), while in Miro1 ΔEF cells there was no significant change (movement was reduced by 23.1% ± 6.9%, p = 0.13).

### Glutamate-Evoked Stopping of Mitochondria Is Associated with Detachment of KIF5 Motors from Mitochondria

To determine whether, for mitochondria in situ, glutamate causes mitochondria to stop by raising [Ca^2+^] and thus detaching Miro1 from KIF motors, we carried out the following experiment. We activated a Ca^2+^ influx through NMDA receptors in brain slices using 100 μM glutamate (with 1 μM glycine) for 10 min and then cross-linked proteins with formaldehyde to preserve protein associations (Vasilescu et al., 2004). We then isolated mitochondria in solution containing 50 μM Ca^2+^ to dissociate motors that were not cross-linked to the mitochondria. (Figure 2I shows, by probing blots of the fractionated tissue for the synaptic vesicle protein SV2 and for cytochrome C, that this procedure produced a satisfactory separation of mitochondria from other small membrane compartments.) This whole procedure was also carried out in parallel on slices without adding glutamate.

Probing the resulting blots for KIF5 motors (and for cytochrome C as a loading control), we found that glutamate application led to a 45% decrease in the amount of KIF5 motor bound to mitochondria (p = 0.008; Figures 2J and 2K). Since glutamate will act predominantly on dendrites, but not all mitochondria in the brain are in the dendrites, glutamate must decrease by more than 45% the number of KIF5 motors that are attached to dendritic mitochondria (e.g., assuming that only 62% of mitochondria are dendritic, as in cat visual cortex [Wong-Riley, 1989] and that the distribution of KIF5 associated with mitochondria before glutamate is applied reflects the distribution of mitochondria, glutamate would have to lower the amount of KIF5 associated with dendritic mitochondria by 89% to produce the overall measured decrease of 45%).

The data presented so far suggest that glutamate evokes mitochondrial stopping via a Ca^2+^-dependent dissociation of Miro1 from KIF5 motors, as shown in Figure 2L. The role of TRAK2 in this mechanism remains uncertain at present, but our data show that TRAK2 binds to Miro1 independently of the Ca^2+^ level, as shown in Figure 2L.

### Activation of Glutamate Receptors Recruits Mitochondria to Synapses

**Figure 4:**
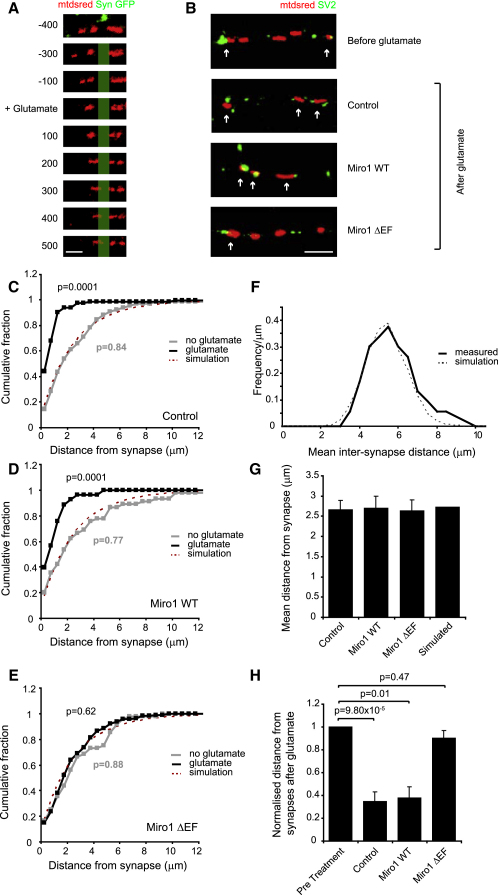
Activation of Glutamate Receptors Recruits Mitochondria to Synapses by a Mechanism Depending on the EF Hands of Miro1 (A) Coculture of neurons expressing either synaptophysin GFP or mtdsred2. Time-lapse microscopy shows that upon glutamate stimulation (starting at time = 0) mitochondria (red), which initially are spread out throughout the process, move to synaptophysin-positive synaptic zones (actual GFP fluorescence is shown in the top panel and is schematized as green bar in lower panels). Scale bar, 2 μm. (B) Neuronal dendrites transfected with mtdsred2 alone, or mtdsred2 plus Miro1 WT, or Miro1 ΔEF before (mtdsred2 alone) and after glutamate treatment. Cells were fixed and stained with the presynaptic marker SV2 (green). Arrows show mitochondria in contact with presynaptic zones. Scale bar, 5 μm. (C) Cumulative distribution of the distance of mitochondria from the nearest synapse in untransfected cells without (gray line, n = 129 mitochondria in 6 dendrites) and with glutamate application (black line, n = 84 mitochondria in 6 dendrites). p values compare measurements with Monte Carlo simulation of random mitochondrial and synaptic distribution in 10,000 dendrites (dotted red line). (D) Cumulative distribution in Miro1 WT-transfected cells without (gray line, n = 45 mitochondria in 4 dendrites) and with glutamate (black line, n = 53 mitochondria in 6 dendrites) compared to Monte Carlo simulation of random mitochondrial and synaptic distribution (dotted line). (E) Cumulative distribution in Miro1 ΔEF-transfected cells without (gray line, n = 48 mitochondria in 4 dendrites) and with glutamate (black line, n = 113 mitochondria in 8 dendrites) compared to Monte-Carlo simulation of random mitochondrial and synaptic distribution (dotted line). (F) Frequency distribution of mean intersynapse distance from measured values (black line, n = 34 dendrites) and simulations (dotted line). (G) Mean distance of mitochondria along dendrite from SV2-positive clusters without glutamate treatment (untransfected controls, n = 213 mitochondria; Miro1 WT, n = 98 mitochondria; Miro1 ΔEF, n = 161 mitochondria, p values compared with the Monte-Carlo simulation with randomly positioned synapses and mitochondria [right bar] are 0.20, 0.31, and 0.15, respectively). (H) Mean distance of mitochondria from SV2-positive clusters after glutamate treatment, normalized to nontreated values, numbers of mitochondria were as follows: control, 218; wild-type, 57; EF hand mutant, 199.

Correct neuronal function requires mitochondria to be actively positioned in areas of energy use and Ca^2+^ buffering. Therefore, we tested whether the glutamate-evoked stopping of mitochondria was correlated with the position of synapses, where NMDA receptor-mediated calcium entry will be high (Noguchi et al., 2005) and could uncouple Miro1 from KIF5, and where ATP use is high due to the need to actively remove ions that enter through synaptic channels. By making cocultures of cells individually expressing either the synaptic marker synaptophysin GFP to label presynaptic terminals, or mtdsred2 to label mitochondria, we could visualize in live cells synaptic contacts onto dendrites containing fluorescently labeled mitochondria (Figure S8). Using this technique, we looked at activity-dependent positioning of mitochondria relative to synapses during glutamate treatment (Figure 4A). Initially, mitochondria were mobile and spread relatively evenly throughout the dendrite. Upon glutamate application, moving mitochondria stopped at areas that were GFP and SV2 positive (Figures 4A and 4B), demonstrating that mitochondria accumulate at synaptic zones. This recruitment of mitochondria to synapses is documented in detail below and in Figures 4C–4H.

To test whether this synaptic recruitment was dependent on Miro1-mediated regulation of mitochondrial transport, we fixed neurons coexpressing Miro1 WT or Miro1 ΔEF together with mtdsred2, or expressing mtdsred2 alone, before and after treatment with 30 μM glutamate and 1 μM glycine for 10 min, and then costained with antibodies to the presynaptic marker SV2 (this was used to define synapse position instead of synaptophysin-GFP because some synapses might be made from neurons that do not express synaptophysin-GFP). We measured the mean intersynapse distance in the cultures (which was 5.70 ± 1.53 μm in 78 dendrites from 30 cells; Figure 4F) and used this, along with the mean mitochondrial density in dendritic processes, to create a Monte Carlo simulation where synapses and mitochondria were randomly positioned along a dendrite at the measured mean densities. In each simulated dendrite, the distance between each mitochondrion and its nearest synapse was calculated. The simulation was run 10,000 times, to produce a distribution of expected values (Figure 4C, dashed line), which predicted the mean value to be 2.58 ± 0.01 μm, assuming that mitochondria are not spatially constrained (Figure 4G). We then measured the mean distance from each mitochondrion along the dendrite to the nearest SV2-positive cluster in each condition and compared these values to the simulation. In both untransfected controls and Miro1 WT cells, no treatment gave mean distances from synapses that were essentially identical to that expected for a random distribution of mitochondria (untransfected controls, 2.66 ± 0.23 μm; Miro1 WT, 2.70 ± 0.47 μm; Figures 4C, 4D, and 4G, p = 0.84 and p = 0.77 compared to the simulation). Upon glutamate application, however, the mean distance between mitochondria and synapses dropped significantly, showing that glutamate receptor activation accumulates mitochondria at synapses (untransfected controls 0.93 ± 0.23 μm, p = 0.0001 compared to nontreated, Miro1 WT 1.01 ± 0.27 μm, p = 0.0001; Figures 4C, 4D, and 4H). In contrast, in Miro1 ΔEF-transfected cells, the mean values stayed within the range expected for a random arrangement, both with and without glutamate (nontreated 2.64 ± 0.42 μm, after glutamate 2.38 ± 0.17 μm, p = 0.88 and p = 0.62, respectively, compared with the simulation; Figures 4E, 4G, and 4H).

### Synaptically Released Glutamate Acts on NMDA Receptors to Produce Miro1-Mediated Stopping of Mitochondria at Synapses

To investigate whether Miro1 activity is also important for regulating mitochondrial movement in response to more physiological increases in neuronal activity, we tested the effect of synaptically released glutamate on the transport of mitochondria in dendrites. Whether activity-dependent alterations in mitochondrial trafficking can occur on a rapid timescale (seconds) has not been reported. To investigate this, we used either electrical field stimulation or treatment with bicuculline (to block GABA_A_ receptor-mediated inhibition) to increase action potential firing and evoke synaptic activity in mtdsred2-transfected cells.

**Figure 5:**
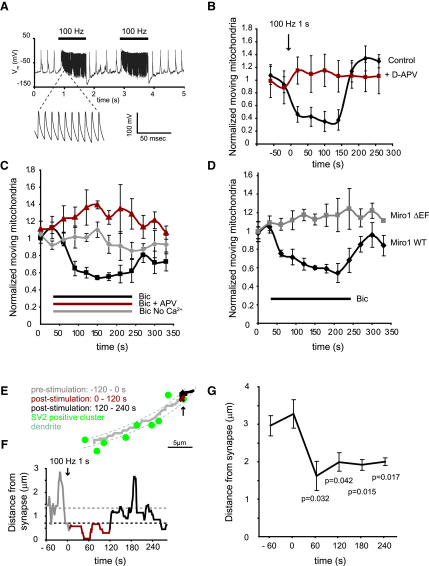
Stimulation of Neuronal Activity Recruits Mitochondria to Synapses as a Result of Ca^2+^ Entering via Synaptic NMDA Receptors Binding to Miro1's EF Hands (A) Field stimulation increases action potential firing in cultured neurons (recorded in current-clamp mode using the whole-cell patch-clamp technique). Voltage recording before, during, and after two 100 Hz stimulation trains: inset shows activity at a faster timescale. (B) Stimulation of cultures at 100 Hz for 1 s results in a rapid drop in mitochondrial movement that lasts for ∼150 s after stimulation (0.35 ± 0.18 of mitochondria moving 120 s after stimulus normalized to baseline, n = 5 dendrites, 53 mitochondria, p = 0.009). This effect is blocked by D-APV (1.15 ± 0.25 fold increase, relative to control, of mitochondria moving after 120 s, n = 4 dendrites, 71 mitochondria, p = 0.14). (C) Neural activity and synaptic glutamate release evoked by block of GABA-mediated inhibition with bicuculline results in increased stopping of mitochondria. Traces are normalized to 2 min baseline movement before the experiment; values stated below are at maximum response at 150 s. Addition of 50 μM bicuculline decreased movement to 0.54 ± 0.08 (n = 6 dendrites, 166 mitochondria, p = 0.0005) of the control level, whereas the same treatment in zero calcium had no effect (1.10 ± 0.09 fold over control, n = 4 dendrites, 193 mitochondria, p = 0.66). Addition of 50 μM D-APV and bicuculline resulted in a slight increase in movement (1.38 ± 0.05 fold over control, n = 4 dendrites, 200 mitochondria, p = 0.03). (D) The bicuculline effect can be blocked using Miro1 ΔEF. Miro1 WT-expressing cells respond in an equivalent manner to control cells to bicuculline treatment (0.59 ± 0.07 of baseline value, n = 4 dendrites, 128 mitochondria, p = 0.006), whereas Miro1 ΔEF-expressing cells do not respond (1.12 ± 0.06, n = 5 dendrites, 226 mitochondria, p = 0.22). (E) Path of a single mitochondrion along a dendrite before (gray) and during and after (red and black at times shown in [F]) a 1 s period of field stimulation at 100 Hz. Synapse SV2 label shown as green. (F) Distance from the nearest synapse at any given time for the mitochondrion in (E). (G) Mean distance of mitochondria from the nearest synapse in the dendrites of five neurons for field stimulation as in (E) and (F).

Electrical stimulation of the coverslip of cultured neurons at 100 Hz for 1 s dramatically increased neuronal firing (measured by voltage recording using whole-cell clamping in current-clamp mode; Figure 5A) and produced a corresponding increase in excitatory synaptic currents (measured in voltage-clamp mode, not shown). This stimulation resulted in a marked reversible decrease in mitochondrial movement that lasted for ∼150 s (the fraction of mitochondria moving after the stimulus was reduced to 35% ± 18% of that moving before the stimulus, p = 0.009; Figure 5B) before returning to baseline levels (presumably when [Ca^2+^]_i_ falls again, although we cannot rule out the possibility that slow reattachment of Miro1 to KIF5 contributes to the duration of the movement inhibition). The fact that the mitochondrial motion eventually returns to control levels shows that the stopping response is not a pathological response to glutamate receptor activation. The stimulation-evoked stopping of mitochondria was blocked by the addition of 50 μM D-APV (Figure 5B), demonstrating that activity-dependent reversible mitochondrial stopping in dendrites is mediated by NMDA receptor activation.

As an alternative approach, we treated neurons with bicuculline, which rapidly increased the frequency of EPSPs, EPSCs, and associated action potentials (Figures S9A–S9D). This resulted in a rapid and consistent drop in mitochondrial transport to 54% ± 8% of the prestimulation value within 120 s (p = 0.0005; Figure 5C). This reduction was blocked by removing extracellular calcium (bicuculline then increased mitochondrial transport by 10% ± 9%, p = 0.66), or in the presence of D-APV (increased by 36% ± 20%, p = 0.3) which significantly decreased the mean inward current induced by the neural activity evoked by bicuculline (Figure S9E). Cells transfected with Miro1 WT showed responses to bicuculline treatment similar to control cells (mitochondrial movement decreased by 59% ± 7%, p = 0.006; Figure 5D). In contrast, Miro1 ΔEF-transfected cells showed no alteration in mitochondrial stopping in response to bicuculline (mitochondrial movement increased by 12% ± 6%, p = 0.22; Figure 5D).

Thus, mitochondrial stopping during neural activity is produced by synaptically released glutamate activating NMDA receptors and evoking the entry of Ca^2+^, which binds to Miro1.

We then assessed whether synaptically evoked activation of NMDA receptors causes mtdsred2-labeled mitochondria to become localized at synapses, as was seen for exogenously applied glutamate in Figure 4 (for this experiment, we only analyzed the positions of mitochondria that were moving, unlike for the experiments of Figure 4, where all mitochondria were measured: see Experimental Procedures). Electrical stimulation of the cultures did indeed result in a similar recruitment of mitochondria to synapses (Figures 5E–5G). Figure 5E shows the path of a single mitochondrion along a dendrite before (gray), during and after (red and black) a 1 s period of stimulation at 100 Hz. The distance from the nearest synapse at any given time is shown in Figure 5F (note that the location of the nearest synapse, identified by the SV2 labeling in Figure 5E, is different at different times). The 1 s period of stimulation caused the mitochondrion to stay close to one particular synapse (arrow in Figure 5E) for about 120 s, before it resumed its normal movement. The mean distance of mitochondria from the nearest synapse in five dendrites from five neurons is shown in Figure 5G: stimulation reduces the mean distance from synapses by ∼40%.

### Miro1-Mediated Recruitment of Mitochondria to Synapses Occurs Even When Only a Few Synapses Are Active

**Figure 6:**
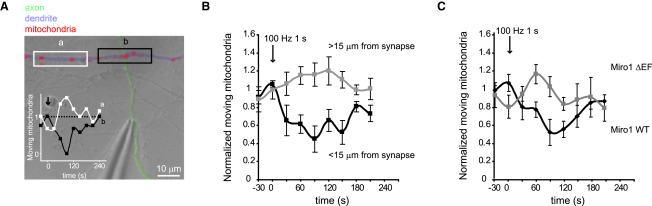
Miro1-Mediated Recruitment of Mitochondria to Synapses Occurs Even When Only a Few Synapses Are Active (A) Image of cell axon (colored green for clarity) stimulated with a pipette to evoke local synaptic input to a dendrite (blue). Mitochondria labeled with mtdsred2 appear red (for clarity, a mask has been used to remove mitochondrial fluorescence from most of the image). Boxes show regions over which mitochondrial motility was measured near (black) and away (white) from the synaptic input. Inset shows normalized percentage mitochondrial movement before, during, and after 100 Hz stimulation at t = 0 (two 1 s trains separated by 2 s) in boxed areas near (black) and away from synaptic input (white). (B) Mean normalized percent mitochondrial movement before, during, and after 100 Hz stimulation at t = 0, near to (black, 5 dendrites, 45 mitochondria) and away from (gray, 5 dendrites, 53 mitochondria) the site of synaptic input. (C) Experiment as in (A) and (B) comparing results at sites within 15 μm of synaptic inputs for cells expressing wild-type Miro1 and the Miro1 EF hand mutant (in addition to mtdsred2). Mean data are from 8 dendrites with 79 mitochondria for Miro1-WT cells and 8 dendrites with 69 mitochondria for Miro1-ΔEF cells.

Next we extended the results to a situation where only a few synapses were activated, by locally stimulating an input to a cell with a patch pipette (Figure 6A). Two 1 s stimulation trains at 100 Hz (with a 2 s gap between the trains) produced a local halting of mtdsred2-labeled mitochondria within a 15 μm region centered on the apparent contact of the input axon with the dendrite (mitochondrial movement was reduced to 45% ± 14% of that before stimulation at 90 s, p = 0.037, n = 5 dendrites; Figure 6B), which lasted ∼150 s, as was found for field stimulation of the cultures (Figure 5B). In contrast, at distances greater than 15 μm from the activated input, no mitochondrial halting occurred (mitochondrial movement increased nonsignificantly by 16% ± 13% [p = 0.3], p = 0.0057 compared to the decrease seen in the region of synaptic input; Figure 6B).

Carrying out the same experiment in neurons that were also transfected with (in addition to mtdsred2) either wild-type Miro1 or the EF hand mutant of Miro1 showed that this recruitment of mitochondria to synapses depended on the Ca^2+^-binding EF hand of Miro1 (Figure 6C). Measuring at 90 s in neurons transfected with Miro1-WT stimulation reduced mitochondrial movement near synapses to 49.1% ± 0.2% of its control value (p = 0.047), while in Miro1-ΔEF-transfected cells there was no change in movement (increased by 3% ± 12%, p = 0.96 compared to before stimulation), a response significantly different from that of Miro1-WT-transfected cells (p = 0.02).

Thus, Miro1-mediated mitochondrial stopping evoked by synaptic NMDA receptor activation can recruit passing mitochondria to active synapses, where energy and calcium buffering demands will be higher, even when only a few synapses are active.

## Discussion

The transport of mitochondria to locations in neurons where ATP is needed to reverse the ion influx generating synaptic and action potentials is critical for neuronal function, but the mechanisms underlying these events have remained elusive. Here we show that in neurons Miro1 links mitochondria to KIF5 motor proteins that transport the mitochondria along microtubules and that this linkage is inhibited by micromolar levels of intracellular Ca^2+^ as a result of Ca^2+^ binding to the EF hand domains of Miro1. This Ca^2+^-dependent linkage allows activation of glutamate receptors to regulate mitochondrial movement and thus to recruit mitochondria to activated synapses.

Our data reveal several interesting features of the mechanism controlling mitochondrial trafficking in dendrites. First, we show that Miro1 is an important regulator of mitochondrial trafficking in neurons and is rate-limiting for mitochondrial transport, since increasing Miro1 expression levels increased the number of moving mitochondria, while knocking down Miro1 had the opposite effect (Figure 1). The effects of altering Miro1 expression were produced through alterations of the fraction of mitochondria that were moving, with no change in the speed of moving mitochondria, suggesting that Miro1 simply attaches mitochondria to moving motor proteins. Indeed, Miro1 binds directly to KIF5 motors (Figure 2A). Second, the calcium-binding Miro1 EF hand domain mutant facilitated mitochondrial movement to the same extent as expressing wild-type Miro1 (Figure 3). This implies that the Miro1 EF hand domains are not necessary for interaction with KIF5 motors per se. Third, the EF hand calcium-sensing domains are, however, critically important for allowing increases in intracellular Ca^2+^ concentration to inhibit mitochondrial movement (Figure 3).

Increased Ca^2+^ levels disrupted Miro1-KIF5 complexes, in a mechanism dependent on the Miro1 EF hand domains. The maximum inhibition of the Miro1-KIF5 interaction was less in the in vitro system with only KIF5 and GST-Miro1 present (Figure 2B) than in brain lysate, where the interaction was almost completely inhibited at high [Ca^2+^] (Figure 2H). This may reflect a modulation of the effect of Ca^2+^ binding by other associated proteins in situ or the presence of competing proteins in the brain lysate to which KIF5 can bind once it is freed from Miro1, and we assume the experiments examining the interaction in brain lysate best reflect the in vivo situation. The IC_50_ for calcium disrupting the Miro1-KIF5 interaction in brain lysate was ∼1 μM (with a K_m_ estimated to be between 0.4 and 2.5 μM, depending on the assumptions made). A similar IC_50_ (∼0.4 μM) for Ca^2+^ inhibiting Miro-mediated facilitation of mitochondrial trafficking was reported in a heart cell line in a recent paper (Saotome et al., 2008), and a simple model based on Ca^2+^ inhibiting the Miro1-KIF5 interaction (see Experimental Procedures) predicts that increasing the level of Miro will decrease the IC_50_ for Ca^2+^ inhibiting mitochondrial trafficking, as was observed experimentally by Saotome et al. (2008). The micromolar IC_50_ value that we measure is substantially above the baseline free [Ca^2+^]_i_ level in neurons (∼50 nM: Maravall et al., 2000), but will be reached at the base of dendritic spines when Ca^2+^ enters through NMDA receptor channels. Noguchi et al. (2005) (Figures 1C, 2E, 3D) report dendritic [Ca^2+^]_i_ rises of 1–6 μM in response to activation of the NMDA receptors on a single dendritic spine, and during repetitive synaptic input the [Ca^2+^]_i_ rise will be correspondingly larger. Thus, the measured IC_50_ has an appropriate value to explain the inhibition of mitochondrial trafficking that we see when we activate neuronal NMDA receptors with synaptically released glutamate (Figures 5 and 6). In addition, a small inhibition of mitochondrial transport is predicted during the dendritic [Ca^2+^]_i_ rise produced by single action potentials (∼250 nM: Maravall et al., 2000).

Glater et al. (2006) proposed that the linkage of mitochondria to KIF motors in *Drosophila* involves dMiro on mitochondria binding to Milton, which in turn binds to kinesin. In contrast, we have shown that mammalian Miro1 can bind directly to KIF5 in a Ca^2+^-dependent manner (Figure 2), which may suggest differences between the mode of attachment of mitochondria to KIF motors in invertebrates and in mammals. In *Drosophila*, Milton was also shown to have a second binding site for mitochondria, suggesting that, for binding of Ca^2+^ to dMiro to detach mitochondria from KIF5 in invertebrates, it must produce conformation changes both in dMiro and in Milton. In mammals, there are several potential candidates for Milton's role, with the closest homolog being GRIF-1/TRAK2 (Brickley et al., 2005) (although the proteins OIP106/TRAK1 and HAP1 also have significant sequence homology [Brickley et al., 2005; Fransson et al., 2006; Stowers et al., 2002], and the kinesin adaptors syntabulin and RanBP2 also have important mitochondrial trafficking functions [Cai et al., 2005; Cho et al., 2007]). We have shown that Miro1 binds to TRAK2 in a Ca^2+^-independent manner (Figure 2D), implying that in mammals Ca^2+^ does not detach mitochondria from KIF motors by acting at the binding site between Miro1 and TRAK2. A full analysis of the Miro1-KIF5 complex will require further work to (1) identify the mammalian homolog (if any) that replaces the invertebrate Milton, (2) establish whether this homolog binds solely to Miro1 or also (like Milton) to mitochondria, and (3) determine how the GTPase domains of Miro1 interact with Ca^2+^ binding to the EF hands to regulate mitochondrial attachment to KIF5 (since Saotome et al. [2008] reported that the GTPase domains are needed for Miro to mediate Ca^2+^-dependent suppression of mitochondrial movement). However, the end result of the glutamate-evoked [Ca^2+^]_i_ rise that stops mitochondrial movement is that KIF5 motors become detached from the mitochondria (Figure 2K).

A different model for how Ca^2+^ and Miro control mitochondrial trafficking was proposed in a recent paper. Wang and Schwarz (2009) suggested that Miro does not interact directly with KIF5, as we have shown in Figure 2, but requires a mammalian homolog of *Drosophila* Milton as an adaptor, although they did report a low level of Miro-KIF association without *Drosophila* Milton present. Since Milton has only 35% similarity to its closest mammalian homolog (TRAK2), the dMilton-mediated potentiation of the coimmunoprecipitation of Miro by KIF observed by Wang and Schwarz (2009) may reflect an interaction specific to dMilton that does not occur in mammals. In addition, Wang and Schwarz (2009) proposed that Ca^2+^ does not detach Miro from KIF5 (as we demonstrate occurs in Figure 2) but instead allows Miro to interact with the motor domain of kinesin, thus preventing the motor from interacting with microtubules. Surprisingly, the [Ca^2+^] concentration needed for this to occur was far higher (50 μM) than we found was necessary to block Miro1-KIF binding (∼1 μM; Figure 2) and much higher than Saotome et al. (2008) and Yi et al. (2004) found was needed to inhibit mitochondrial movement (∼0.4 μM). Further work is needed to resolve these important mechanistic differences.

The Ca^2+^-dependent inhibition of the Miro1-KIF5 interaction that we have characterized allows mitochondria to be uncoupled from motor proteins and thus to accumulate in microdomains of increased calcium concentration within the cell. This behavior is evoked at the postsynaptic side of synapses by exogenously applied or synaptically released glutamate (Figures 4–6) and might also occur at the presynaptic side when action potentials open presynaptic calcium channels, although we have not investigated this. Thus, the probable functional role, during neuronal activity, of the regulation of mitochondrial movement by Miro1 is to locate mitochondria near the postsynaptic membrane where they are needed to provide calcium buffering and to provide ATP to pump out ions that enter through synaptic channels.

We have shown that mitochondria are recruited to active synapses even when only a few synapses are active (Figures 6A and 6B). Further work is needed to determine the minimum duration of synaptic activity needed for effective glutamatergic control of mitochondrial motion. Large rises of [Ca^2+^]_i_ during excitotoxic situations also cause mitochondrial movement to cease (Rintoul et al., 2003), suggesting that the pathway we report here is also relevant to pathological conditions.

## Experimental Procedures

### Antibodies, Constructs, and Reagents

The SUK4 function-blocking hybridoma was obtained from the Developmental Studies Hybridoma Bank. Mitochondrially targeted dsred fluorescent protein (mtdsred2) was from Clontech. GFP and myc-tagged human Miro1 constructs and the Miro1 antibody used for immunoprecipitations have been described previously (Fransson et al., 2006). A second antibody raised against Miro1 was used for western blotting and was from Sigma-Aldrich. Antibodies against cytochrome C and mtHsp70 were from Santa Cruz. shRNAi were targeted against rat Miro1 and were expressed from the pSUPER.GFP.neo vector (Oligoengine), Miro1 RNAi1 sequence was 5′-TGGGAACAAATCTGACCT-3′, RNAi2 was 5′-AATGCCTCCGCCTCAAGCC-3′, while the scrambled control was 5′-GGAATCTTCCTGCTTTGGG-3′.

### Cell Culture

Low-density cultures of hippocampal neurons were prepared from postnatal day 0 rats as described previously with minor modifications (Darcy et al., 2006; Banker and Goslin, 1991). Coverslips were coated by spraying 5 μg/ml poly-D-lysine (Sigma) and 0.4 mg/ml rat tail collagen. Astrocytes were preplated with culture media consisting of BME (Invitrogen), 10% fetal-calf serum (Invitrogen), 0.15% glucose (Sigma), 10 mM HEPES (pH 7.35), 10 mM sodium pyruvate, and penicillin/streptomycin (BD Biosciences). After 3–7 days, dissociated neurons were plated on astrocytes in neurobasal medium (Invitrogen) containing 10% fetal bovine serum, 0.15% glucose, and 2 mM glutamine. At 1 or 2 days in vitro (DIV), media were exchanged to neurobasal medium (Invitrogen) containing 5% fetal-calf serum (Invitrogen), 0.15% glucose, 2 mM glutamine, 10 mM KCl, and penicillin/streptomycin. 4 μM cytosine-arabinoside (Sigma) was added to culture media at 2 DIV. Neurons were used for experiments at 8–14 DIV. Animal care and use were according to guidelines of the United Kingdom Home Office. For all experiments, except where stated otherwise, neurons were transfected at DIV 6–8 using a calcium phosphate method as described (Xia et al., 1996). To quantify RNAi knockdown of Miro1, shRNAi constructs were transfected using Amaxa nucleofection, which has a transfection efficiency of 50%–60% (Kittler et al., 2004, 2006). For experiments coculturing two populations of neurons transfected with either mtdsred2 or GFP-synaptophysin, transfection was by Amaxa nucleofection, and the two sets of cells were mixed before plating. For antibody transduction of neurons, affinity-purified SUK4 or 9E10 antibody as a control were transduced across the plasma membrane at DIV 10–14 using Chariot reagent (Active Motif) following the manufacturer's instructions with minor modifications (Kittler et al., 2006; Morris et al., 2001; Payne et al., 2003; Remacle et al., 2005; Sloane and Vartanian, 2007). In brief, a 100 μl mix of 2 μl of Chariot reagent complexed with 10 μg of antibody was overlaid onto cultured neurons with 100 μl serum-free media and incubated at 37°C. After 1 hr, another 100 μl of serum-free media was added and the preparation incubated for a further 3 hr before imaging.

### In Vitro GST Pull-Down

GST-fusion proteins were produced in *E. coli* and purified as described previously (Kittler et al., 2006). KIF5A, KIF5B, KIF5C, TRAK2 (amino acids 1–700) and TRAK2 (amino acids 476–700) were in vitro translated (IVT) from their appropriate pRK5 vectors using TNT SP6 Quick coupled Transcription/Translation system (Promega) and labeled with [^35^S]-methionine (Amersham Biosciences) following the manufacturer's instructions with a 50 μl final volume. 2.5 μl of ^35^S labeled IVT protein was incubated for 1–2 hr at 4°C with 20 μg of fusion protein immobilized on glutathione sepharose beads (or GST alone as control) in a final volume of 500 μl diluted in buffer (50 mM HEPES, 125 mM NaCl, 5 mM H-EDTA, 1 mM PMSF, and antipain, pepstatin, and leupeptin at 10 μg/ml). Complexes were washed three times in buffer by pelleting at 1000 rpm and resuspension in 1 ml of buffer. Finally the beads were resuspended in 10 μl of 3X sample buffer and resolved by SDS-PAGE. An input of ^35^S-IVT corresponding to 10% of that in the experiment was run in parallel. Gels were dried and radioactivity detected using a phosphor storage screen. ^35^S-IVT protein binding to fusion proteins was normalized to ^35^S-IVT protein input and binding to GST alone subtracted before results were expressed as a percentage of binding in the absence of calcium.

### Immunoprecipitation and GST Pull-Down from Brain Lysate

Brain lysate (5 mg of total protein) was solubilized in a buffer containing 50 mM HEPES, 125 mM NaCl, 1% Triton, 5 mM EDTA, 1 mM PMSF, and antipain, pepstatin, and leupeptin at 10 μg/ml. Solubilized material was ultracentrifuged and the supernatant (solubilized protein) exposed to 4 μg anti-Miro or control IgG with or without 2 mM [Ca^2+^]_free_ (Palmer et al., 2005) followed by precipitation by protein A Sepharose. For GST pull-down experiments, GST-fusion proteins were produced in *E. coli* and purified as described above. Thirty micrograms of GST fusion proteins coupled to Sepharose beads were added to 5 mg brain lysate as above with varying levels of [Ca^2+^]_free_ as shown. [Ca^2+^]_free_ was varied by adding an amount of CaCl_2_ calculated using the site http://www.stanford.edu/∼cpatton/webmaxc/webmaxcS.htm. Bound material was washed five times in the above buffer before elution with SDS sample buffer. Bound material was then western blotted using bioreactor concentrated anti-SUK4 (1:20) antibody and anti-Miro antibody (1:500) and ECL. HRP-conjugated anti-mouse and anti-rabbit secondary antibodies for western blotting were from Rockland and used at 1:5000. Densitometric quantification of bands on gels was carried out using a Biorad GS-800 calibrated densitometer.

### Mitochondrial Isolation

Mitochondria were isolated from COS7 cells as described previously (Frezza et al., 2007).

For mitochondrial isolation after stimulation, 150 μm whole-brain slices were cut from P12 rats in artificial cerebrospinal fluid (ACSF) (126 mM NaCl, 24 mM NaHCO_3_, 1 mM NaH_2_PO_4_, 2.5 mM KCl, 2.5 mM CaCl_2_, 2 mM MgCl_2_, and 10 mM glucose) containing kynurenic acid (1 mM) at room temperature and gassed with 95% O_2_/5% CO_2_. Slices were allowed to recover for 1 hr before kynurenic acid was removed by placing the slices in ACSF for 1 hr.

ACSF containing 1 μM glycine and 100 μM glutamate at 37°C was applied to slices for 10 min to activate synaptic NMDA receptors. Slices were then immediately fixed in 1% formaldehyde for 10 min (Vasilescu et al., 2004) and the reaction quenched in 125 mM glycine for 5 min.

Mitochondria were then isolated as previously described (Frezza et al., 2007). Briefly, slices were homogenized in isolation solution (10 mM Tris/MOPS, 1 mM EGTA/Tris, 20 mM sucrose, pH 7.4, [Ca^2+^] set to 50 μM by adding an amount of CaCl_2_ calculated using the site http://www.stanford.edu/∼cpatton/webmaxc/webmaxcS.htm), centrifuged at 600 × g for 10 min, and the supernatant centrifuged at 7000 × g for 10 min to pellet mitochondria. The pellet was washed in isolation buffer and dissolved in 1% SDS. Protein concentration was determined using a BCA protein assay (Pierce). Cross-links were reversed by boiling in SDS for 30 min, and samples were resolved by gel electrophoresis. Bands on gels were quantified as above.

### Calcium Overlay Assay

Calcium binding was examined using a ^45^Ca^2+^ overlay assay (Maruyama and Nonomura, 1984). Briefly, GST-fusion proteins were produced in *E. coli* and purified as above and deposited on a nitrocellulose membrane using a Bio-Dot SF Microflitration Apparatus (Biorad). Membranes were then stained with Ponceau-S to visualize protein loading. Membranes were washed for 10 min in PBS to remove Ponceau-S before being washed three times in wash buffer (10 mM imidazole HCl, 60 mM KCl, and 5 mM MgCl_2_, pH 6.8). Membranes were then incubated with 5 μCi/ml of ^45^CaCl_2_ in wash buffer for 30 min at room temperature and rinsed for 2 min in distilled H_2_O followed by 30 s rinse in 50% ethanol. Membranes were thoroughly dried at room temperature and exposed to a phosphorimaging screen for 48–96 hr.

### Live Imaging

Cells were imaged under perfusion with extracellular solution pH 7.4 (125 mM NaCl, 5 mM KCl, 10 mM HEPES, 10 mM glucose, 2 mM CaCl_2_, and 1 mM MgCl_2_). In Ca^2+^-free experiments, Ca^2+^ was omitted from the buffer. Working solutions were prepared from the following stock solutions: 100 mM glutamate in water, 1 M glycine in water, 100 mM APV in water, 100 mM bicuculline in water. Medium was warmed to 37°C and flowed at a rate of 5 ml min^−1^ throughout the duration of each experiment. For acquisition, we used a Zeiss Pascal upright confocal microscope and LSM software with a Achroplan 63× water-immersion lens with 0.95 numerical aperture. Images were acquired at 1 frame/s unless stated otherwise. Excitation was via a HeNe laser at λ = 543 nm and an argon laser at λ = 488 nm.

### Image Processing

Mitochondrial movements were analyzed in neuronal dendrites. Dendrites and axons can be clearly distinguished: each mtdsred2-labeled axon in the low-density cultures we use can be clearly identified as a long thin process that extends for many hundreds of microns (at least three to five times longer than dendrites) while the dendrites are far shorter, thicker, and have a different branching morphology (Figure S2). Confirmation of the identity of dendrites was obtained by labeling with antibody to the protein MAP2 (Figure S2), which is expressed in dendrites but not axons (Caceres et al., 1984).

Mitochondrial mobility was assessed by counting the percentage of mitochondria moving during an imaging period (2 min unless stated otherwise). Mitochondria in a field of view were classed as moving if they moved more than 2 μm in 2 min. For bicuculline and electrical stimulation experiments, images were split into 20 or 30 s bins and analyzed as above for each bin over the course of the experiment. For glutamate and FPL 64176 treatments, pretreatment analysis was −3 min to −1 min, and posttreatment was 10 min to 12 min.

Kymographs were created as described previously (Kang et al., 2008; Miller and Sheetz, 2004). Briefly, the “LSM reader” macro was used to open Zeiss images in imageJ. Curved processes were straightened using the “straighten” macro and kymographs created by the “multiple kymograph” macro. Resultant kymographs show the process along the x axis and time across the y axis. Height of the kymographs is 2 min unless otherwise stated, and time increases down the page.

Mitochondrial velocity was measured using the “track points” macro of Metamorph (Universal Imaging). Images were taken every second. The smallest change of position measurable was one pixel, i.e., ∼0.3 μm. Periods of no movement within a track for 3 s or greater were discounted from analysis. The resulting coordinates were used to calculate the average velocity (μm s^−1^) of all mobile mitochondria in each condition.

For the synaptic recruitment experiments in Figures 5E–5G and 6, mitochondrial tracks were created using the track points macro of Metamorph. To allow us to quantify the rapid changes of mitochondrial localization evoked by brief stimulation, we only analyzed the positions of mitochondria that moved more than 2 μm throughout the 400 s duration of the experiment, with the aim of avoiding the data being dominated by mitochondria that did not move throughout the experiment. Resultant coordinates were superimposed onto images of the same postfixed cells stained for SV2. Monte Carlo simulations and the distance from the center of each mitochondrion to the position at the middle of the dendrite opposite the nearest SV2-positive cluster were calculated using custom programs written in Mathematica (Wolfram Research).

### Electrophysiology

Cells were superfused at 29°C ± 1°C with solution containing (mM) 125 NaCl, 5 KCl, 10 HEPES, 10 glucose, 2 CaCl_2_, and 1 MgCl_2_, pH set to 7.4 with NaOH. Neurons were whole-cell patch-clamped with pipettes (resistance in bath 6–7 MΩ, series resistance in whole-cell mode 8–20 MΩ) containing (mM) 130 K-gluconate, 4 NaCl, 10 HEPES, 10 EGTA, 0.5 CaCl_2_, 0.5 Na_2_GTP, 4 MgATP, pH set to 7.3 with KOH. For voltage-clamp experiments where bicuculline was applied, 5 mM QX-314 was added to the intracellular solution. Junction potentials were compensated. Drugs were applied in the superfusate. Field stimulation was applied using two parallel platinum wires mounted at each side of the bath. Local stimulation employed a patch pipette (resistance ∼1 MΩ) filled with external solution. Recordings were made using an Axon MultiClamp 700B amplifier and analyzed in Clampfit (both Molecular Devices, USA).

### Statistical Analysis

All data were obtained using cells from at least three different preparations. Numbers of cells studied are given in the text or figure legends. Statistical significance across groups was analyzed using a one-way ANOVA. Individual differences were assessed using individual student's t tests. In cases where multiple comparisons were made within a single experiment, p values were adjusted using the Bonferroni method, where p is the p value obtained by the student's t test multiplied by *k*, the number of paired comparisons. Data are shown as mean ± SEM.

### Predicted Effect of [Miro] on IC_50_ for Ca^2+^ Inhibiting Mitochondrial Movement

We assume that mitochondria can exist in three states: not bound to Miro and therefore stationary (the fraction of mitochondria in this state, A, is denoted *a* below), bound to Miro (and hence to KIF5) and moving along microtubules (state B, with fraction *b* in this state), and bound to Miro but with Ca^2+^ bound to (a single site on) Miro as well, so the mitochondria are stopped (state C, fraction *c*). This produces the reaction scheme

**Figure:**
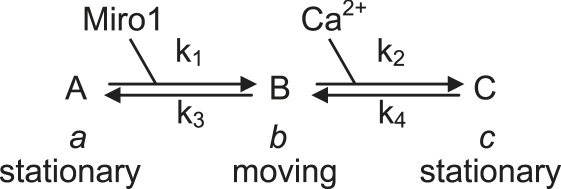

where K_Miro_ = k_3_/k_1_ is the binding constant for Miro1 binding to mitochondria, and K_Ca_ = k_4_/k_2_ is the binding constant for Ca^2+^ binding to Miro1. Solving the equilibrium equations for this scheme, gives the fraction of mitochondria that are moving (i.e., (*b*/(*a + b + c*)) as

$$\text{fraction}\;\text{moving}\;\text{=}\frac{\text{1}}{1+\frac{{\text{K}}_{\text{Miro}}}{\left[\text{Miro}\right]}+\frac{\left[{\text{Ca}}^{2+}\right]}{{\text{K}}_{\text{Ca}}}}$$

This equation predicts that the effective K_m_ for Ca^2+^ inhibiting mitochondrial movement is K_effective_ = K_Ca_.{1 + (K_Miro_/[Miro])}. Thus, the effective K_m_ value is predicted to be reduced when extra Miro is expressed (down to a minimum value of K_Ca_ at very high Miro levels), as was observed by Saotome et al. (2008) for a heart cell line.

## Supplemental Data

Document S1. Supplemental Data

Movie S1 Time-lapse imaging of hippocampal neurons transfected with mtdsred2 and Miro1 GFP. Images were acquired every 5 s. Miro1 GFP and mtdsred2 are almost completely colocalized in mitochondria.

Movie S2 Glutamate induced stopping of mitochondria in hippocampal dendrites. Neurons were transfected with mtdsred2 and imaged before and after glutamate treatment. Images were acquired every 2 s. Imaging periods were −3 to −1 min and 10−12 min relative to glutamate treatment. Scale bar, 10 μm.

Movie S3 Glutamate treatment does not inhibit all organelle transport. Processes transfected with synaptophysin GFP were imaged before and after glutamate treatment. Images were acquired every 5 s. Imaging periods were −3 to −1 min and 10−12 min relative to glutamate treatment. Scale bar, 5 μm.

Movie S4 Glutamate induced stopping of mitochondria in hippocampal dendrite transfected with Miro1 WT. Neurons were transfected with mtdsred2 and Miro1 and imaged before and after glutamate treatment. Images were acquired every 2 s. Imaging periods were −3 to −1 min and 10–12 min relative to glutamate treatment. Scale bar, 10 μm.

Movie S5 Miro1 ΔEF uncouples glutamate-induced stopping of mitochondria in hippocampal dendrites. Neurons were transfected with mtdsred2 and Miro1 ΔEF and imaged before and after glutamate treatment. Images were acquired every 2 s. Imaging periods were −3 to −1 min and 10–12 min relative to glutamate treatment. Scale bar, 10 μm.
