# Supplementary material for: Miro1 Is a Calcium Sensor for Glutamate Receptor-Dependent Localization of Mitochondria at Synapses
Source: Neuron. 2009 Feb 26;61(4):541–55. doi: 10.1016/j.neuron.2009.01.030 (PMC2670979; doi:10.1016/j.neuron.2009.01.030)
Supplement: Document S1. Supplemental Data [file mmc1.pdf]

NEURON, VOLUME 61

SUPPLEMENTAL DATA

MIRO1 IS A CALCIUM SENSOR FOR GLUTAMATE RECEPTOR DEPENDENT LOCALIZATION OF MITOCHONDRIA AT SYNAPSES

Andrew F. MacAskill[1], Johanne E. Rinholm[1*], Alison E. Twelvetrees[1*], I. Lorena Arancibia-Carcamo[1], James Muir[1], Asa Fransson[2], Pontus Aspenstrom[2], David Attwell[1] and Josef T. Kittler[1]

SUPPLEMENTARY INFORMATION

Includes:

Supplementary Figures 1 – 9

Supplementary Fig.1 Mitochondrial localization of Miro1 in hippocampal neurons.

(A) Static image of a dendrite at time = 0 in a cell with mitochondria labelled with TMRM. (A') Kymograph showing mitochondrial movement in the dendrite. Height 2 min (time increases down the page), scale bar 20 μm.

(B) Percentage of mitochondria that were moving over a 2 min period in 11 dendrites with mitochondria labelled with TMRM (182 mitochondria were studied) and 8 dendrites with mitochondria labelled by expression of mitochondrially targeted mtdsred2 (295 mitochondria were studied: same data as in Fig. 1D).

(C) Hippocampal neurons were transfected with Miro1 GFP and a mitochondrial marker mtdsred2, 2 – 3 days before being imaged at DIV 12. Scale bar 20 μm; rectangle in right panel shows area expanded in D.

(D) Miro1 GFP shows almost complete overlap with mitochondria in neuronal processes. Scale bar 10 μm.

(E-F) Miro is present in both moving and stationary mitochondria. (E) and (F) show still images of processes at time 0. Kymographs (E', F') show mitochondria in process as line scan across the X axis, and time over the Y axis (time increases down the page). Vertical lines represent stationary mitochondria and diagonal lines represent moving mitochondria. Miro1 GFP (E, E') and mitochondria (F, F') in neuronal processes show co localization over time. Images were acquired every 5 sec. Vertical scale bar 1 min, horizontal scale bar 10 μm.

Supplementary Fig 2. Identification of dendrites in sparse hippocampal culture.

(A-C) Composite image showing hippocampal cell in sparse culture transfected with mtdsred2. (A) MAP 2 labeling. (B) mtdsred2. (C) Overlay of MAP2 and mtdsred2 labeling. Sparse culture allows short dendrites with elongated mitochondria to be easily

distinguished from axons, which are 3-5 times longer with shorter, more rounded mitochondria and a much more sparse mitochondrial occupancy. (D) Schematic showing axon/dendrites in this cell as identified by morphology/MAP2 analysis. Scale bar 20 μm.

Supplementary Fig. 3. Effect on mitochondrial movement of knock-down of Miro1, and rescue by expressing shRNAi-resistant Miro1

(A, B, C, D) Static image of dendrites in mtdsred2 transfected neurons (at time = 0) which are also transfected with (A) Miro1 RNAi, (B) another Miro1 RNAi (targeting a sequence different from that in A, this is the same RNAi as is used in Fig. 1 of the main text), (C) scrambled Miro1 RNAi, and (D) the RNAi used in B plus RNAi-resistant Miro1.

(A', B', C', D') Kymographs showing decreased mitochondrial movement in neuronal dendrite on shRNAi expression (A', B') compared to scrambled control (C'), and rescue by expression of RNAi-resistant Miro1 (D'). Height 2 min (time increases down the page), scale bar 10 μm.

(E) Expression level of Miro1 on expressing scrambled RNAi, and the two different RNAi constructs, with actin level as a loading control.

(F) Percentage of mitochondria moving in dendrites of control cells (n=8, 259 mitochondria), cells expressing two types of RNAi to knock-down Miro1 (n=8 dendrites, 356 mitochondria for RNAi1 and n=18 dendrites, 842 mitochondria for RNAi 2), cells expressing a scrambled control RNAi (n=9 dendrites, 429 mitochondria), and cells expressing RNAi plus Miro1 (n=6 dendrites, 525 mitochondria). P values compare with the scrambled bar.

Supplementary Fig. 4. Miro1 speeds mitochondrial movement both towards and away from the soma

Percentage of moving mitochondria normalized to untransfected control cells in anterograde (away from soma) and retrograde (towards soma) directions caused by transfection of Miro1 WT and Miro1 ΔEF. Miro1 WT and Miro1 ΔEF cause equal increases in mitochondrial movement in both directions.

Supplementary Fig. 5. Characterization of antibodies and antibody transduction

(A, B) Transduction of SUK4 antibody (A) and 9E10 control antibody (B) using Chariot reagent effectively introduces antibody into cultured neurons identified by GluR1 staining (i), but this does not occur if we omit the antibody (ii), the Chariot reagent (iii), or both (iv). The red channel was imaged with pinhole at maximum to allow detection of transduced antibody. Scale bar 10 μm

(C) A Sigma Miro1 antibody specifically recognizes Miro1 GFP transfected into COS7 cell lines.

(D) The Miro1 antibody originally characterized in Fransson et al. (2003), successfully immunoprecipitates Miro1 GFP transfected into COS7 cells.

Supplementary Fig. 6. Glutamate dependent alterations in mitochondrial motility are mediated by calcium. Neurons were transfected with mtdsred2 and imaged before and after treatment with 30 μM glutamate and 1 μM glycine for 10 minutes with and without calcium in the extracellular medium.

(A-C) Kymographs (A'-C') showing mitochondrial movement before (A) and after glutamate treatment without calcium (B) and with calcium (C) present in the extracellular medium. Height 2 min, scale bar 10 μm

(D) Percentage of mitochondria moving before and after treatment with glutamate, with calcium in the extracellular medium (n=4 dendrites, 202 mitochondria) and without calcium in the extracellular medium (n=4 dendrites, 226 mitochondria). Values were normalized to the percentage of moving mitochondria before treatment, and p values are for comparison with this.

(E) Percentage of mitochondria moving after treatment with 50mM KCl and the L-type calcium channel agonist FPL64176, normalized to percentage moving before treatment for dendrites of untransfected (n=5 dendrites, 462 mitochondria), Miro1 WT (n=6 dendrites, 431 mitochondria), and Miro1 ΔEF (n=5 dendrites, 391 mitochondria) cells.

(F-H) Miro1 binds calcium in a manner occluded by mutating its EF hand domains (Miro1 ΔEF). (F) $^{45}Ca^{2+}$ overlay of GST-PICK1 (as a positive Ca^{2+}-binding control: Hanley & Henley, 2005), GST-Miro1-WT and GST alone (as a negative control). (G) $^{45}Ca^{2+}$ overlay of GST Miro1 WT and GST Miro1 ΔEF. (H) Values in graph are totals with GST alone subtracted, normalized to WT binding. GST fusion proteins were slot-blotted onto a nitrocellulose membrane and exposed to $^{45}Ca^{2+}$. GST Miro1 WT binds $^{45}Ca^{2+}$, whereas for GST Miro1 ΔEF this is significantly impaired (0.56 ± 0.12, n=4, p=0.0017). Equal protein loading is visualized using Ponceau stain.

Supplementary Fig. 7. Miro1 mediated effects on trafficking are mitochondrial specific and glutamate treatment does not inhibit all organelle transport.

(A-B') Processes transfected with synaptophysin GFP. Kymographs (A'-B'') show movement of synaptophysin GFP positive vesicles before (A', B') and after (A'', B'') treatment with 30 μM glutamate and 1 μM glycine for 10 minutes.

(C) Percentage of synaptophysin GFP vesicles moving before and after glutamate treatment (n=6 dendrites, 229 vesicles).

Supplementary Fig. 8. Example of co-culture of neurons transfected separately with synaptophysin GFP and mtdsred2.

Typical field of view with synaptophysin (green) positive axons contacting mitochondria (red) – filled dendrite. Arrows show points of contact of dendrites with axons.

Supplementary Fig. 9. Electrophysiological response of cultured neurons to bicuculline

(A-C): Application of 50 μM bicuculline increases action potential firing (recorded in current-clamp mode using the whole-cell patch-clamp technique).

(A) Voltage recording before and during application of bicuculline: insets show EPSPs and synaptic activity at a faster time scale.

(B) Frequency of action potentials in A.

(C) Normalized frequency of action potentials before (control) and during application of bicuculline in 3 cells.

(D) Application of 50 μM bicuculline increases the frequency of EPSCs (recorded at -52 mV in voltage-clamp mode). Pipettes contained QX-314 to block voltage-sensitive Na^+ channels.

(E) Normalized inward current before application of bicuculline (control) and in the presence of bicuculline before, during and after application of 50 µM D-APV, in 4 cells. The values were averaged over at least 1 minute, starting 30 sec after application of bicuculline, 30 sec after application of APV and 2 minutes after removal of APV, respectively. APV significantly decreased the inward current evoked by bicuculline.

References

Fransson, A., Ruusala, A., and Aspenstrom, P. (2003). Atypical Rho GTPases have roles in mitochondrial homeostasis and apoptosis. J Biol Chem *278*, 6495-6502.

Hanley, J.G., and Henley, J.M. (2005) PICK1 is a calcium-sensor for NMDA-induced AMPA receptor trafficking. EMBO J. 24, 3266-3278.

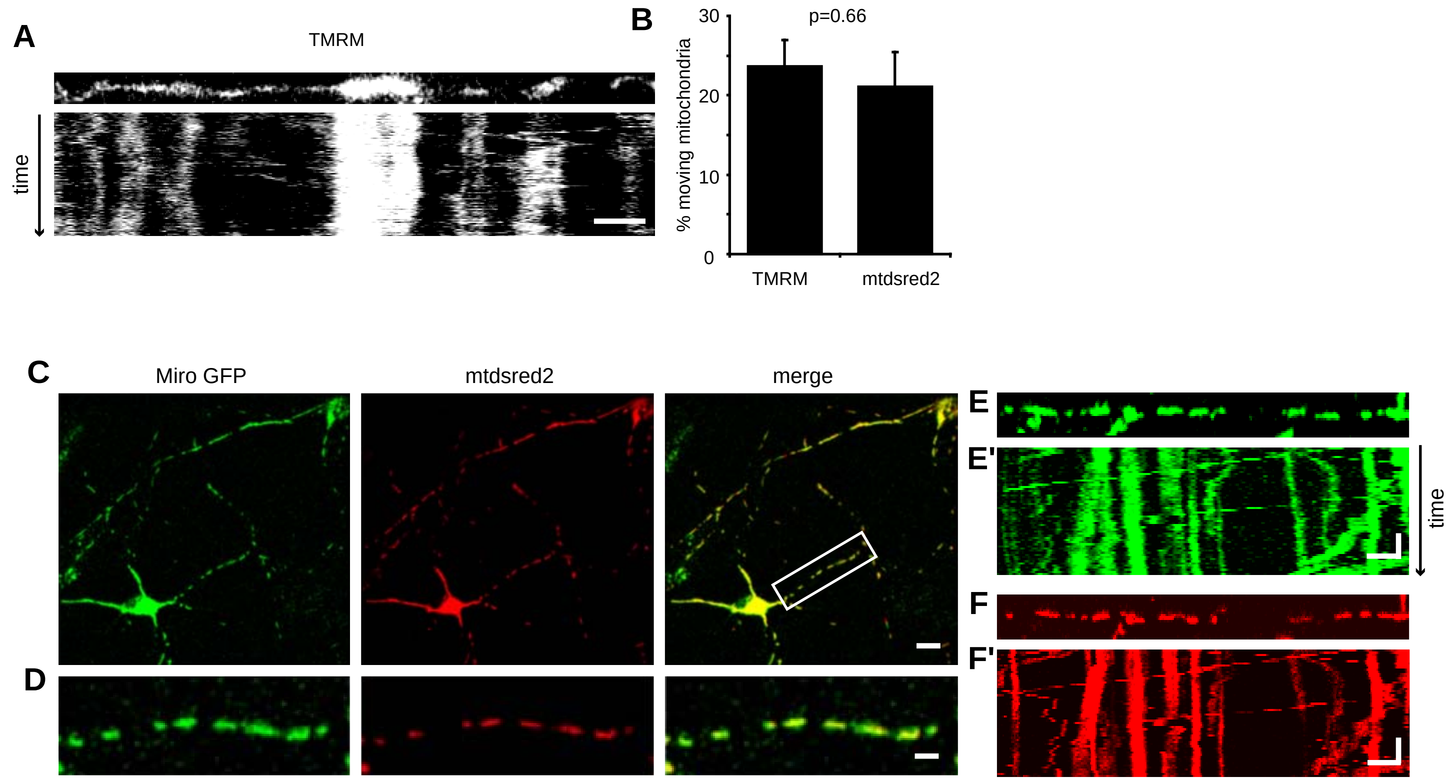

Supplementary Figure 1

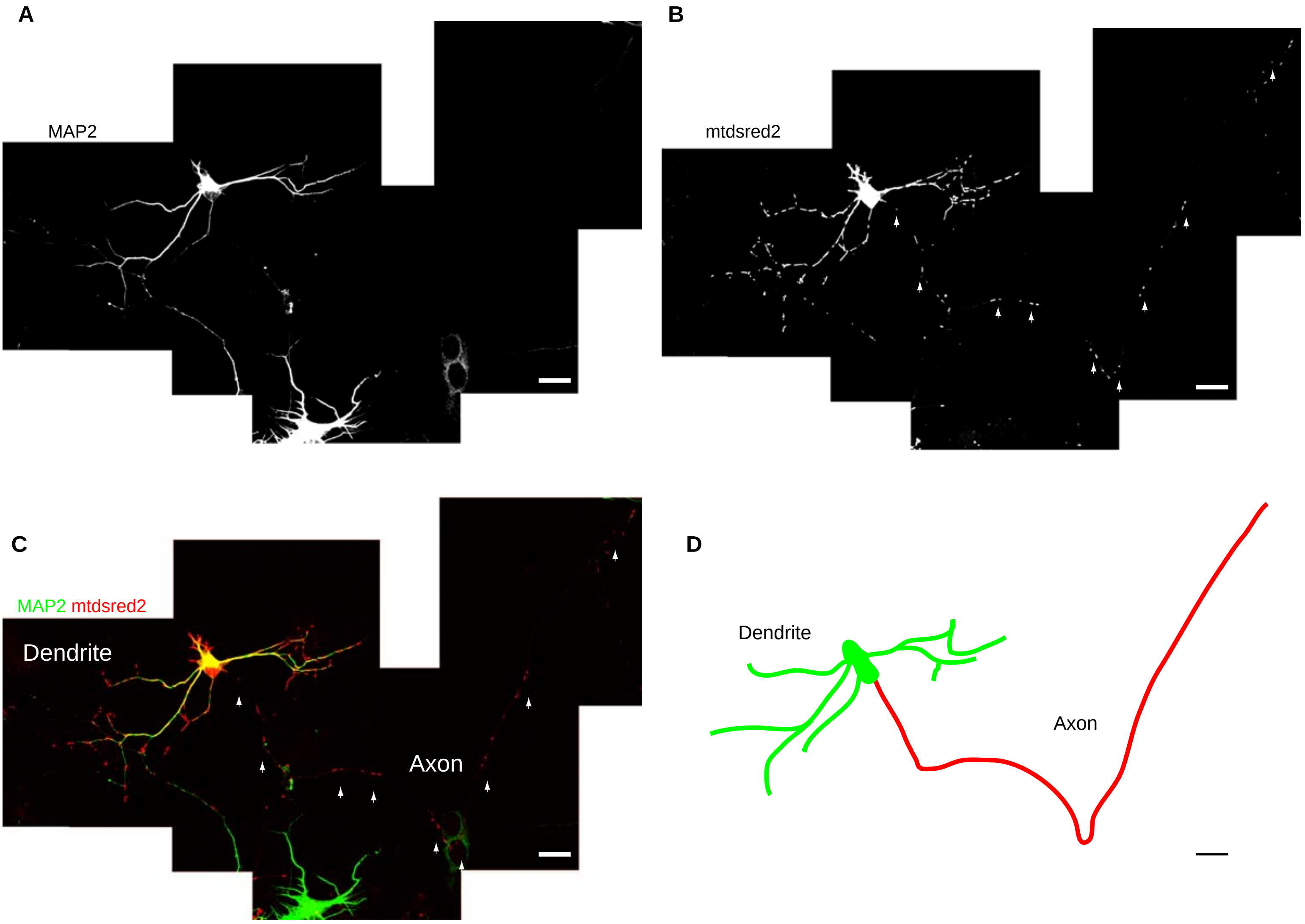

Supplementary Figure 2

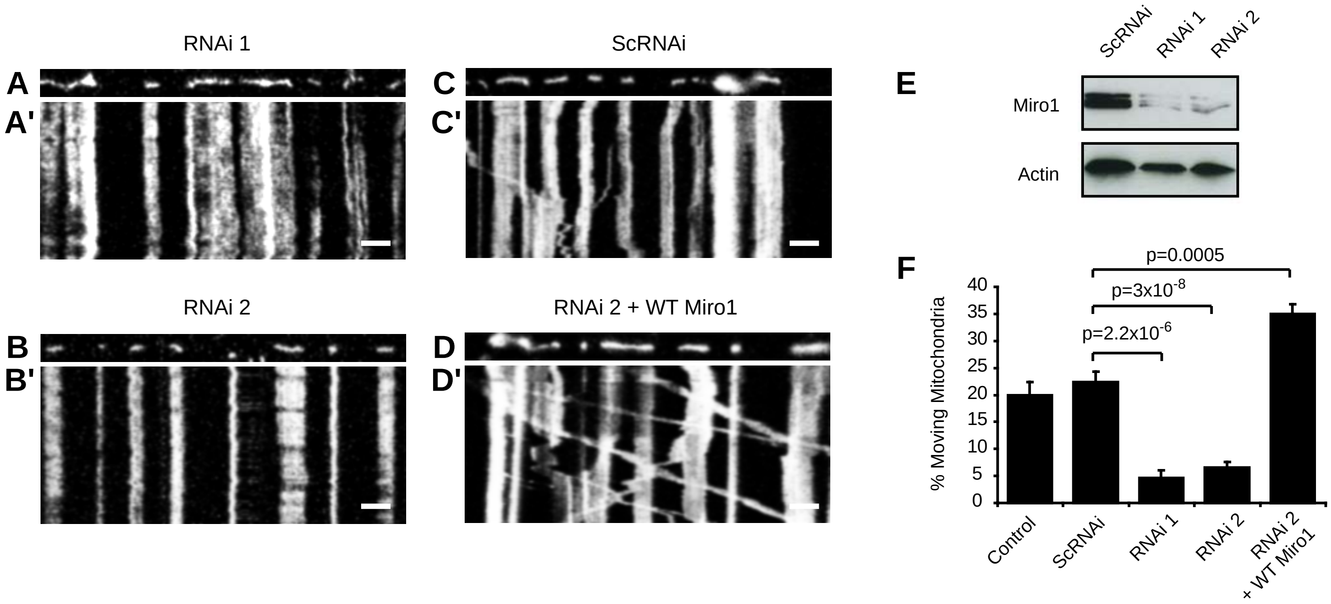

Supplementary Figure 3

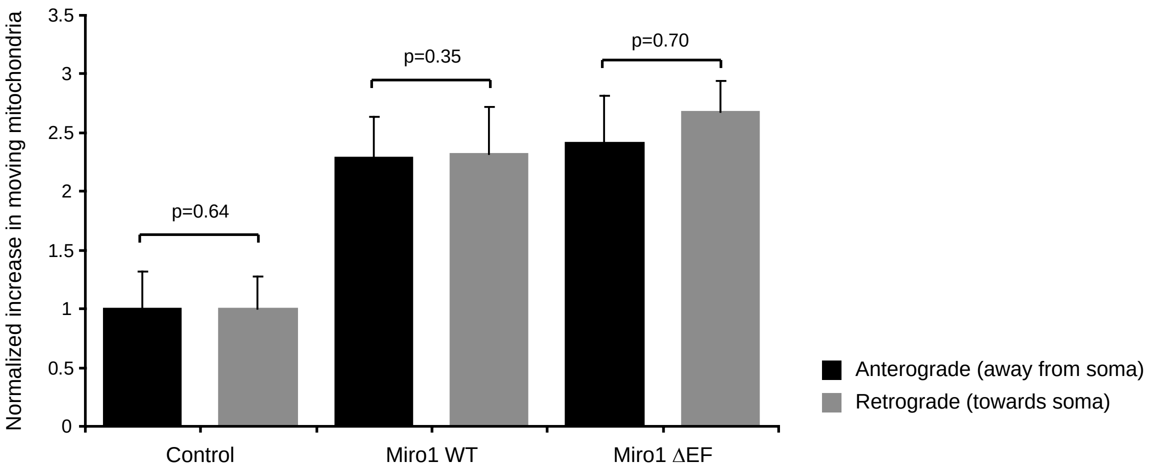

Supplementary Figure 4

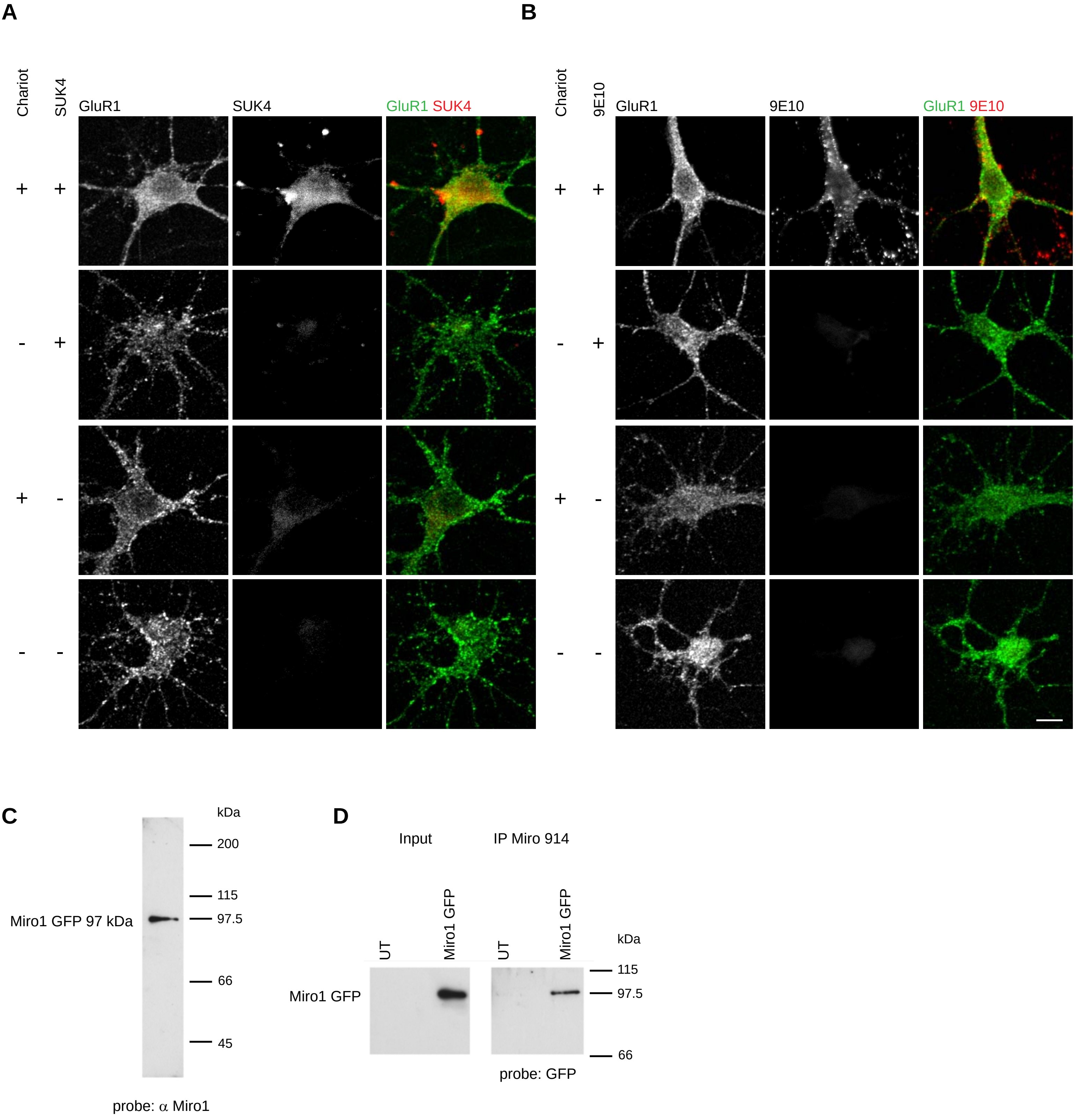

Supplementary Figure 5

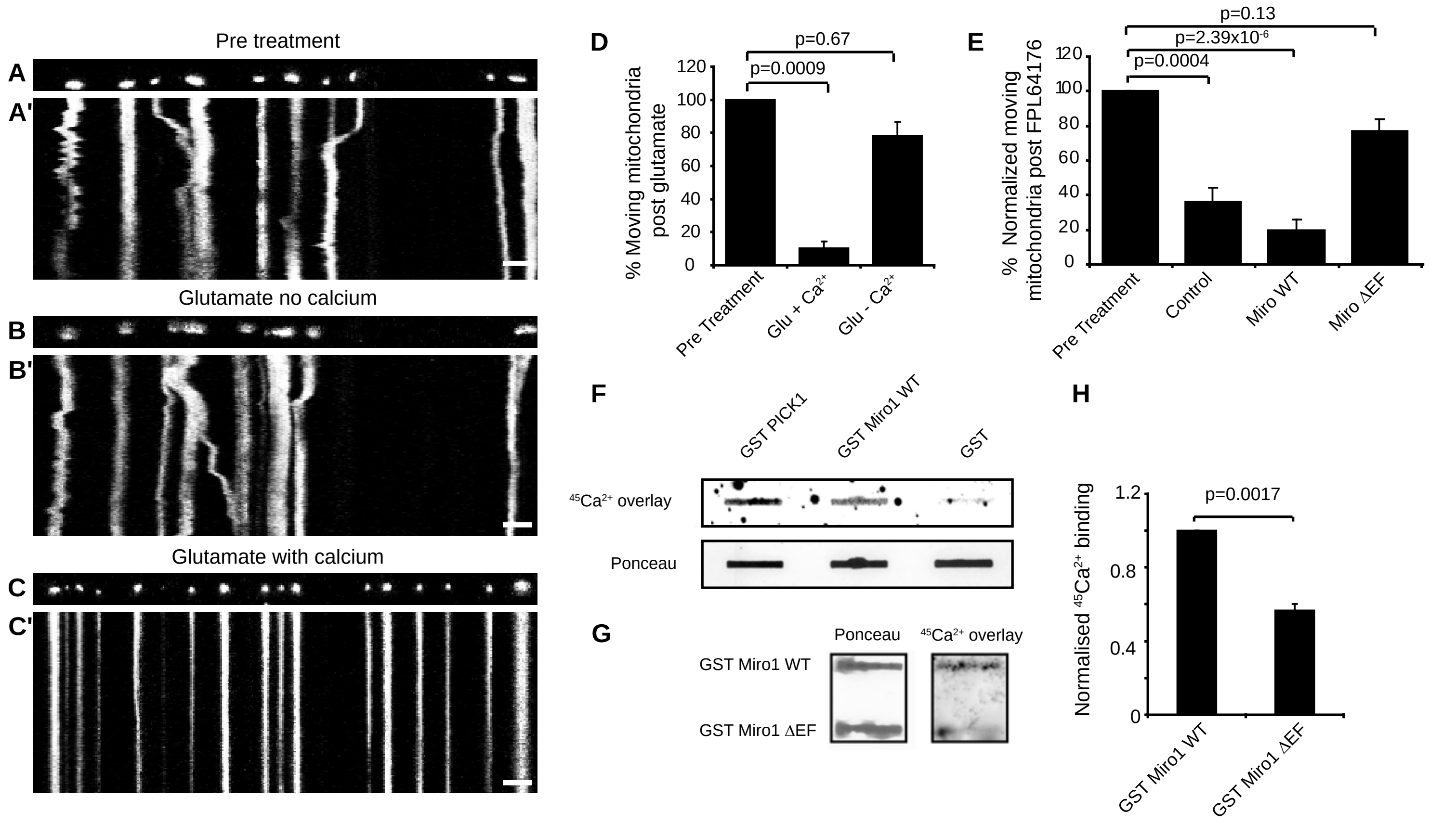

Supplementary Figure 6

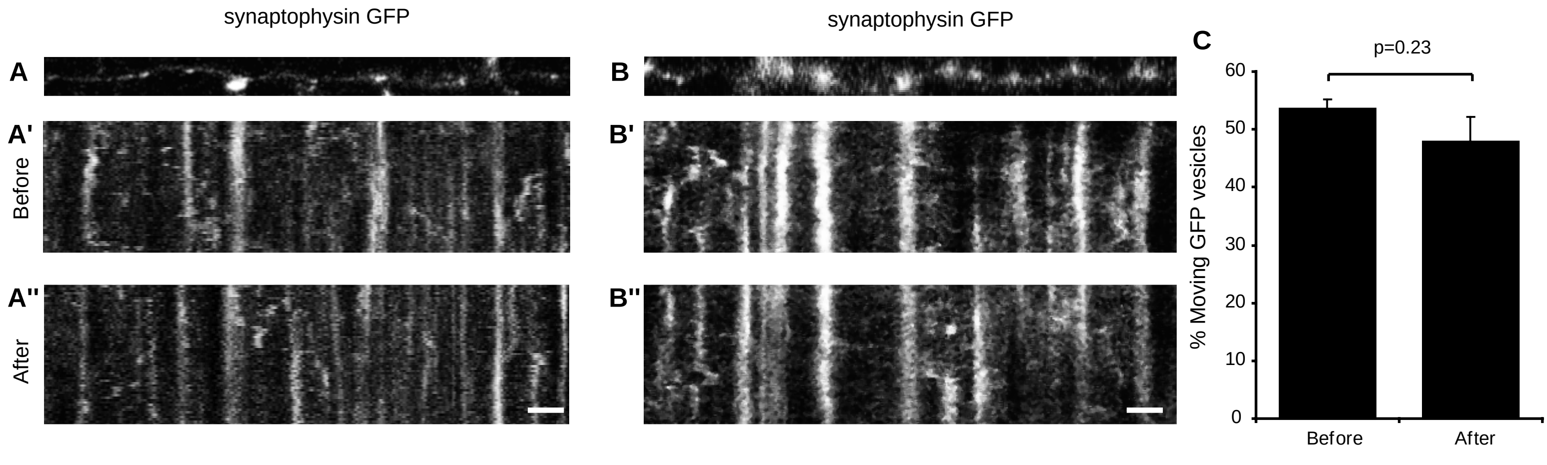

Supplementary Figure 7

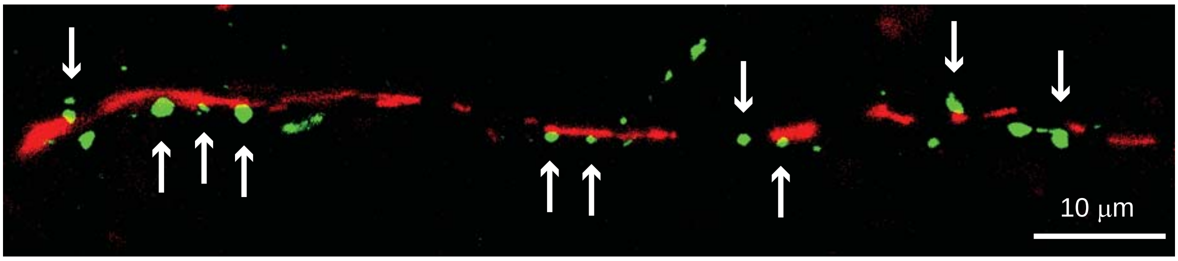

Supplementary Figure 8

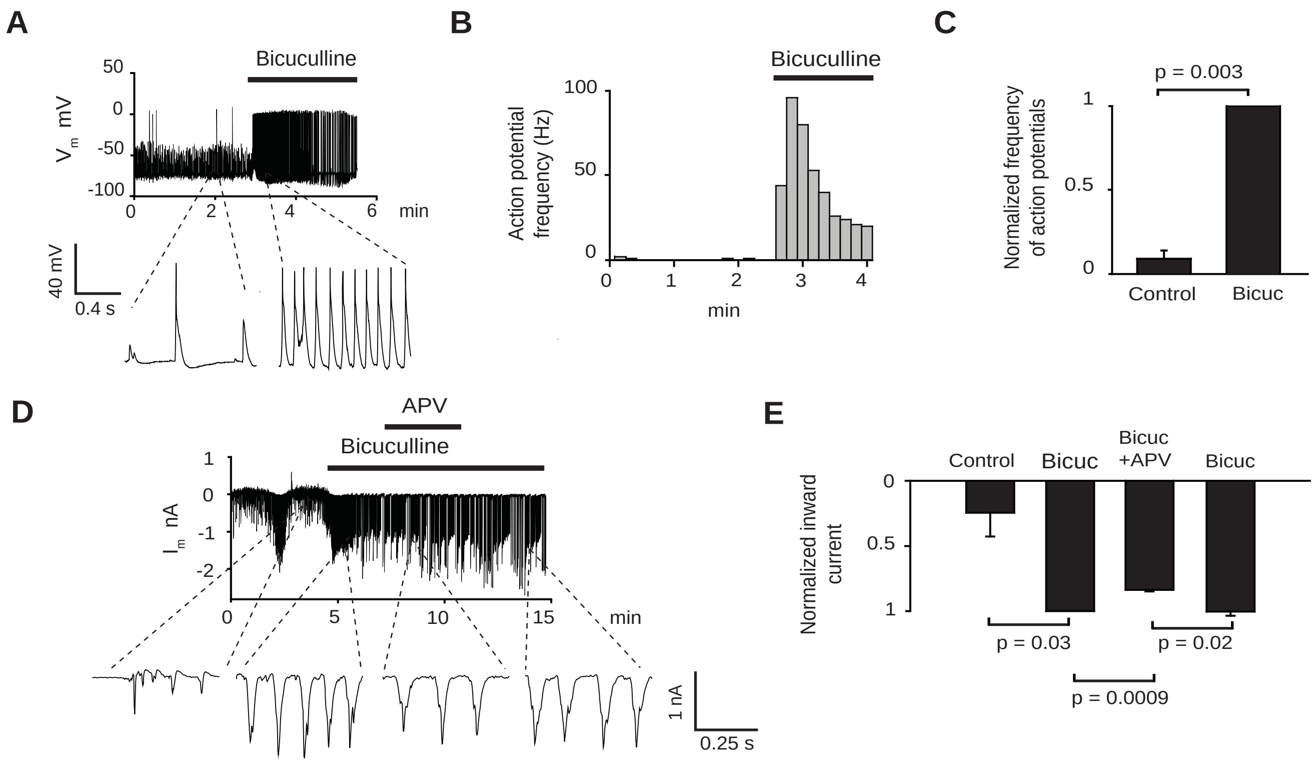

Supplementary Figure 9
